# Yield responses of arable crops to liming – An evaluation of relationships between yields and soil pH from a long-term liming experiment

**DOI:** 10.1016/j.eja.2019.02.016

**Published:** 2019-04

**Authors:** J.E. Holland, P.J. White, M.J. Glendining, K.W.T. Goulding, S.P. McGrath

**Affiliations:** a The James Hutton Institute, Dundee, DD2 5DA, UK; b Department of Computational and Analytical Sciences, Rothamsted Research, Harpenden, Hertfordshire, AL5 2JQ, UK; c Department of Sustainable Agriculture Sciences, Rothamsted Research, Harpenden, Hertfordshire, AL5 2JQ, UK

**Keywords:** Crop-soil interactions, Soil acidity, Crop yield response function, Long-term experiment

## Abstract

The management of optimal soil pH is fundamental to sustainable crop production. Understanding the lime requirement for arable crops has developed gradually over the last several decades. The aim of this study was to examine the yield-pH relationship for a range of arable crops to understand their response to liming, based on the Long-Term Liming experiments established in 1962 at Rothamsted Research, UK. The main treatments of four different rates of lime and, therefore, four distinctly different soil pH levels were maintained for 35 years at two sites (Rothamsted and Woburn). The pH ranged from 4.4 to 8.0. The lime response was tested on the following crops: spring barley, spring oats, spring beans, spring lupins, winter lupins, potatoes, linseed, winter oilseed rape, winter triticale and winter wheat. Relative yield (*RY*) was used for non-linear regression analysis to detect site, year and phosphorus (P) fertiliser effects on the relationship with pH. Liming had a highly significant positive effect on soil pH, but overall there was no consistent increase or decrease in soil extractable P (Olsen) or exchangeable K. There were significant site effects detected for *RY* for most crops which reflect differences in the two soil types. Spring oats and potatoes had very weak responses to lime within the pH range tested. For spring barley, winter triticale, winter wheat and winter oilseed rape significant effects of P fertiliser on the yield-pH relationship were found, although the nature of effects differed between crops and sites. Findings from the Long-Term Liming experiment are invaluable in improving the fundamental understanding on the yield-pH relationship for important arable crops and this has significant implications on selecting crops for rotations. The pH at 90% *RY* was calculated for selected crops and the beneficial effect of fertiliser P was detected in significantly reducing the critical pH value.

## Highlights

• Relative yield increased with greater pH for most crops except potatoes and oats.
• The different yield-pH relationships of crops have implications for arable rotations.
• Soil type significantly effects the nature of the relative yield-pH relationship.
• Long-term liming increases pH, but effects on soil extractable P were inconsistent.
• P fertiliser significantly reduces the critical pH value of selected crops.

## 1 Introduction

At a global scale soils are increasingly being degraded and becoming marginal for agricultural production driven by e.g. salinization, erosion and acidification (FAO, 2015). The principles of soil acidification are well understood (Bolan et al., 2003), but its extent and implications need regular reviewing. Changes to atmospheric nutrient inputs make estimating soil acidification difficult. In the UK there has been a substantial decline in total sulphur (S) deposition over the past 40 years (RoTAP, 2012). For example, the S deposition at the Woburn Farm, Bedfordshire, UK is <5 kg^−1^ ha^−1^ year compared with 85 kg in 1970 (Goulding, 2015). The recent reduction in atmospheric acidic load in the UK has been significant, but uncertainty remains about other acidifying inputs and processes at finer scales. At the farm scale fertilisers exert a fine scale acidifying pressure, e.g. when the long-term application of ammonium-based fertilisers acidify the soil (Goulding, 2016; Johnston et al., 1986). Acidification induced by fertilisers has been observed globally and it is a serious problem in China (Guo et al., 2010). The removal of nutrients via harvested biomass or grain is also an acidifying process (Goulding and Blake, 1998), increasing as yields increase. With all these challenges there is a need to understand the management of soil acidity better.

Liming is a common and long-established management practice to maintain an optimal soil pH for crop production (Goulding, 2015). For most arable crops there is a positive yield response associated with liming. However, there are distinct differences between crops in yield response to lime (Cifu et al., 2004) and crop varieties can differ in their tolerance to acidic soil conditions, e.g. to Al^3+^ (Slattery and Coventry, 1993). Previous studies have quantified the yield-soil pH relationship for several arable crops (Farhoodi and Coventry, 2008; Liu et al., 2004; Slattery and Coventry, 1993), but for many soil types and climatic regions this relationship is not known. Losses of lime impact soil chemical properties. For example, there is a decrease in exchangeable Ca and estimates of the CaCO_3_ losses have been calculated (Bolton, 1977; Chambers and Garwood, 1998). Depending on the source of lime, liming can increase Mg^2+^ relative to Ca^2+^ (Cifu et al., 2004). This type of change is stronger in the surface soil than the subsoil. Liming changes the availability of phosphorus (P) (Haynes, 1982) and this has implications on plant P uptake after liming. There are several other positive and negative effects from liming on soils and crops (Holland et al., 2018). The nature of the crop yield-soil pH relationship has major implications for the sustainability and efficiency of crop production. Unfortunately, there have been an insufficient number of studies which have quantified this relationship and hence there remains a lack of understanding on liming impacts. We have therefore used one of the few long-term experiments that study soil acidification and liming to improve understanding of what is a global problem.

The background to the Long-Term Liming (LTL) experiment begins with the first applications of lime to the Park Grass experiment in 1881 (e-RA, 2017). This was implemented in response to the acidifying effects of some of the fertilisers applied, in particular ammonium salts. Further regular applications of lime to Park Grass during the end of the nineteenth and first half of the twentieth century led to distinctly different soil pH values developing on the different fertiliser treatments by the late 1950s (Warren and Johnston, 1964). During this period interest in amending soil pH with lime and the effects of liming on soils and crops increased as the effects of soil acidity on soils and crops were further investigated (Mann and Barnes, 1940). By the early 1960s interest in liming was increasing, yet Park Grass was the only ‘Classical’ long-term experiment at Rothamsted which included a liming treatment. Consequently, in 1962 a new liming experiment was established at Rothamsted and Woburn farms on sites that had previously received no lime and were acidic (Bolton, 1971).

Long-term experiments have greatly improved understanding of crop and soil management over the past decades, e.g. research findings from Rothamsted have provided significant insights on agricultural sustainability and soil fertility (Johnston and Poulton, 2018). The principal aim of this paper is to quantify the crop yield-soil pH relationship for several arable crops commonly grown in the UK. The objectives were to:

(i) quantify the effect of liming on crop production using a non-linear regression approach by determining the crop yield-soil pH relationship for a range of major arable crops;
(ii) test the effects of soil type on the crop lime response;
(iii) investigate the effect of supplying other nutrient (P, K) treatments on crop yield-soil pH relationship.

## 2 Materials and methods

### 2.1 Experimental site description

The Rothamsted site was located in Sawyers field at Rothamsted Research, Harpenden, Hertfordshire, UK (51.8157 N, 0.3752 W). The soil has a silty clay loam texture. It is classified as Batcombe Series (Bolton, 1977); according to an international soil classification system this corresponds to a Profundic Chromic Endostagnic Luvisol (WRB, 2006). The Woburn site was located in Stackyard field, section-C, at Woburn Experimental Farm, Husborne Crawley, Bedford, UK (52.0003 N, 0.6149 W). The soil at Woburn is a complex of different deposits and the soil texture is a sandy loam. It is classified as Cottenham Series (Bolton, 1977) and it is described as a Eutric Rubic Arenosol (WRB, 2006), although a detailed soil survey shows part of the site is classified as the Stackyard soil series (Catt et al., 1980). Bolton (1977) reports that the Rothamsted soil has greater clay (20 vs. 12% for Rothamsted and Woburn, respectively) and silt content (52 vs. 17% for Rothamsted and Woburn, respectively), while the Woburn soil is sandier (71 vs. 28% for Woburn and Rothamsted, respectively). Additional data and further discussion on the soil properties is available for Rothamsted (Avery and Catt, 1995) and Woburn (Catt et al., 1980).

The sites were cropped from 1962 until 1996; nine different crop types were grown: cereals (barley, oats, triticale, wheat), break or minor crops (linseed, beans, lupins, oil seed rape) and tuber crops (potatoes). Both spring and winter crops were grown, although the majority were spring crops. The same crops were grown at each site. Over the whole experiment there were four fallow years (1969, 1979, 1980, 1984). There were also five years when crops failed at one or both sites for a variety of reasons. For example, in 1976 due to the lack of rainfall at both sites there was no spring oilseed rape seed harvested; in 1990 at Woburn the crop established poorly because of bird damage and in 1994 there was poor winter survival of winter lupins at Rothamsted, while in the same year there was excessive grazing (bird damage) at Woburn. From 1962 until 1996 there were 24 years when crop yield data were available from both sites. For some years no plot level data were available (e.g. 1962 at both sites) and consequently there were data for 52 site years in total. Table 1 presents cropping details for each year of the experiment including the crop type, crop variety and the respective sowing and harvest dates for the Rothamsted and Woburn sites.

**Table 1:** The arable cropping history with the crop type, variety, sowing and harvest dates for each harvest year of the long-term liming experiment at the Rothamsted and Woburn sites 1962–1996.

Year	Crop	Variety	Rothamsted Sowing date	Harvest date	Woburn Sowing date	Harvest date
1962	Spring beans	Tick 30B	16/03/1962	20/09/1962	19/03/1962	20/09/1962
1963	Spring beans	Tick 30B	08/04/1963	18/10/1963	27/03/1963	21/09/1963
1964	Spring beans	Spring Tick	06/03/1964	25/08/1964	13/03/1964	25/08/1964
1965	Spring barley	Maris Badger	17/03/1965	05/09/1965	29/03/1965	28/08/1965
1966	Spring barley	Maris Badger	14/03/1966	26/08/1966	11/03/1966	08/09/1966
1967	Spring barley	Maris Badger	03/03/1967	22/08/1967	04/03/1967	21/08/1967
1968	Potatoes	Majestic	04/04/1968	03/10/1968	29/03/1968	02/10/1968
1969	Fallow	–	–	–	–	–
1970	Spring barley	Julia	28/03/1970	15/08/1970	26/03/1970	12/08/1970
1971	Spring barley	Julia	10/03/1971	16/08/1971	17/03/1971	17/08/1971
1972	Spring barley	Julia	20/03/1972	24/08/1972	15/03/1972	15/08/1972
1973	Spring barley	Julia	12/03/1973	10/08/1973	12/03/1973	13/08/1973
1974	Potatoes	Pentland crown	24/04/1974	30/10/1974	17/04/1974	30/09/1974
1975	Spring oats	Manod	25/03/1975	18/08/1975	20/03/1975	18/08/1975
1976^a^	Spring OSR^b^	Maris Haplona	26/03/1976	14/07/1976	31/03/1976	07/07/1976
1977	Spring oats	Manod	04/04/1977	05/09/1977	31/03/1977	03/09/1977
1978	Spring barley	Porthos	19/04/1978	08/09/1978	15/03/1978	23/08/1978
1979	Fallow	–	–	–	–	–
1980	Fallow	–	–	–	–	–
1981	Spring oats	Peniarth	13/04/1981	10/09/1981	09/04/1981	03/09/1981
1982	Spring oats	Peniarth	14/04/1982	26/08/1982	29/03/1982	20/08/1982
1983	Potatoes	Pentland Crown	23/05/1983	28/10/1983	11/05/1983	07/11/1983
1984	Fallow	–	–	–	–	–
1985	Spring barley	Klaxon	18/03/1985	23/08/1985	18/03/1985	28/08/1985
1986	Winter Triticale	Lasko	23/10/1985	10/09/1986	22/10/1985	07/09/1986
1987	Spring lupins	Vladimir	31/03/1987	17/11/1987	06/04/1987	18/11/1987
1988	Linseed	Anatares	13/04/1988	24/10/1988	22/04/1988	01/11/1988
1989	Spring beans	Alfred	30/03/1989	14/08/1989	31/03/1989	22/08/1989
1990^c^	Spring beans	Alfred	06/03/1990	15/08/1990	05/03/1990	–
1991	Winter OSR^b^	Libravo	31/08/1990	07/08/1991	30/08/1990	13/08/1991
1992^d^	Winter OSR^b^	Libravo	05/09/1991	–	06/09/1991	–
1993^c^	Winter lupins	CH304/70	07/10/1992	10/10/1993	02/10/1992	–
1994^d^	Winter lupins	CH304/70	20/10/1993	–	24/09/1993	–
1995	Winter wheat	Genesis	30/09/1994	02/08/1995	30/09/1994	04/08/1995
1996	Winter wheat	Hereward	28/09/1995	09/08/1996	03/10/1995	19/08/1996

a 1976 harvested as green crop (whole crop) and some plots failed.

b OSR = oilseed rape.

c The crop failed at the Woburn site only.

d The crop failed at both sites.

The agronomy and management of the crops followed conventional practices over the course of the experiment and was the same at both sites. In most years nitrogen (N) fertiliser was applied to crops at a rate appropriate to the crop and site, and a range of conventional pesticides were used to control weeds, diseases and insect pests. All of the information about the experiment is available in the Rothamsted Electronic Archive (e-RA, 2017).

### 2.2 Experimental design

A factorial experimental design was used at each site with two randomised blocks of 16 plots split into two sub-plots. Overall, the experiment applied a total of seven different treatment factors at the plot level; a maximum of four treatment factors were applied in a given year (Table S1). There were four levels of limestone applied (as ground chalk, CaCO_3_) and these are described as zero or control, low (L), medium (M) and high (H). The lime requirement was determined by the methods of Woodruff (1948) and Shoemaker et al. (1961). Over the course of the experiment lime was applied six times. Table 2 shows the total amounts applied and the application dates. Bolton (1977) describes the content and particle size of the limestone applied.

**Table 2:** The dates when lime was applied and the corresponding four rates (control, low (L), medium (M) and high (H) of lime as ground chalk (CaCO3, t-1 ha) applied at Rothamsted and Woburn.

Rothamsted Application dates	Lime rates (t ha^−1^) (Control, L, M, H)	Woburn Application dates	Lime rates (t ha^−1^) (Control, L, M, H)
5 March 1962	0, 5, 10, 15	9 March 1962	0, 5, 10, 15
4 December 1962	0, 0, 0, 5	19 October 1962	0, 0, 2, 4
29 November 1978	0, 2, 5, 10	21 November 1978	0, 1, 2, 4
3-7 December 1981	0, 2, 5, 10	25 November 1981	0, 2, 5, 10
26 November 1982	0, 5, 3, 10	4 November 1982	0, 0, 5, 10
13 November 1986	0, 1, 1.5, 2.5	13 November 1986	0, 1, 1.5, 2.5
Total	0, 15, 24.5, 52.5	Total	0, 9, 25.5, 45.5

The lime treatments were combined with a range of additional nutrient treatments (Table S1). These varied in type (e.g. seed inoculant or fertiliser type and/ or amount) and number during the course of the experiment. For instance, there were tests of a range of nutrients (P, K, Mg, Mn, S) at two or more levels, in selected years e.g. Mn was applied for four years from 1987 to 1990. The lime, phosphorus (P) and potassium (K) treatments were applied to whole plots, while magnesium (Mg), manganese (Mn), sulphur (S) and seed inoculum were only applied to sub-plots. P fertiliser was applied as superphosphate with the amounts applied given in Table 3. K was applied as muriate of potash from 1962 until 1978 as two treatments: 0 (control) and 125 kg K ha^−1^ (+K), except in 1968 when the + K treatment was 188 kg K ha^−1^. The whole plot treatment factors described above were applied to 16 field plots per block with two replicate blocks. The design was a randomised complete block (RCB) from 1962 to 1973. The size of each plot was 6 × 16 m (˜0.01 ha). In selected years from 1974 onwards each whole plot was split into two sub-plots and a sub-plot treatment applied as in Table S1.

**Table 3:** The phosphate (P_2_O_5_) treatments applied with the corresponding amounts (kg ha^−1^) at Rothamsted and Woburn.

Harvest year^a^	P_2_O_5_ applied (kg ha^−1^)
1962-1978^b^	control (0), +P (63)
1968, 1974^c^	control (0), +P (125)
1980^d^, ^e^	control (0), P1 (25), P2 (25), P3 (75)
1981	control (0), P1 (50), P2 (0), P3 (50)
1982 (Rothamsted)	control (0), P1 (0), P2, (50), P3 (50)
1982 (Woburn)	control (0), P1 (50), P2 (50), P3 (100)
1987^c^	control (0), P1 (25), P2 (25), P3 (75)

a P applied in the autumn, except in 1968 and 1974.

b No P applied in 1969 (a fallow year or in the other fallow years: 1979, 1980, 1984).

c For potato crops in 1968 and 1974 only was there a different + P treatment amount applied.

d Residual P from1983 to 1986 and after 1984; no P fertiliser applied in these years.

e From 1980 onwards the two P treatments (control, +P) were divided into four P treatments (control, P1, P2, P3). The control developed into a new control and P1 treatment, and the + P became P2 and P3.

### 2.3 Field measurements and laboratory analysis

Samples were collected from the topsoil (0–23 cm depth) in the autumn/winter after harvest and before sowing the next crop in most years, but there were several years when none were collected. Soil pH was measured in 1: 2.5 soil: water suspensions using a standard electrode and pH meter. Soil chemical properties such as exchangeable cations (extracted with 1 M ammonium acetate adjusted to pH 7) and extractable soil P (Olsen, 1954) were also measured in selected years. Crop grain yields have been standardised and are reported at 85 percent dry matter; oilseeds (linseed and oilseed rape) are expressed at 90 percent dry matter. Potato yields are reported on a fresh weight basis. Further details on the field sampling and soil sample analysis is available (Bolton, 1971, 1977; e-RA, 2017).

### 2.4 Climate

It is well established that climate has a significant influence on crop performance. The two experimental sites are approximately 30 km apart and so there were small differences in the weather between the sites. The mean (1962–1996) annual rainfall (mm) at Rothamsted was 693 and at Woburn it was 638. Rainfall differences over the growing season (April-July) were minimal; at Rothamsted it was 210 mm, while it was 208 mm at Woburn. Nevertheless, during the course of the experiment from 1962 until 1996 there were large differences between the years in key climate variables. The total annual and growing season rainfall, temperature and solar radiation are given for each year for each experimental site in Supplementary Tables S2, S3 and S4. The cumulative total air temperature was calculated from the mean daily air temperature with a base temperature of 0 °C (e-RA, 2017).

### 2.5 Statistical analysis

Analysis of variance (ANOVA) was used to test the soil pH and other soil properties (in particular extractable (Olsen) P and exchangeable K) for significant main and sub-plot treatment effects. At both sites for most years there was plot level soil pH data. Soil measurements were not made at the Rothamsted site in ten of the years and nine of the years at the Woburn site (not consecutive). Plot level data for other soil properties was analysed in a small number of selected years. For instance, there were eight years with extractable P data at Rothamsted and six years at Woburn. In addition, at both sites exchangeable K was determined only in a limited number of years. For years when soil measurements were not made soil pH values were derived by interpolation between established values from the nearest years.

Crop yield effects for each site and year were tested for main and sub-plot treatments using analysis of variance (ANOVA). For each crop type the following effects were tested: lime, P, K (main plots). The other sub-plot treatments (i.e. Mg, Mn, S and seed inoculum) are not reported here. Overall, there were very few significant yield effects among the subplot treatments (data not shown), hence this paper focuses upon the main plot treatments.

Nonlinear regression analysis was applied to investigate the strength and nature of the relationship between harvested yield and soil pH. Due to seasonal and site differences it was considered appropriate to use relative yield (*RY*) to express the effect of liming for a crop response (Dyson and Conyers, 2013). Here the *RY* is defined as the ratio of the actual yield (*Y*) to the measured maximum yield (*Y_m_*) for a given crop in a specific year and site (i.e. *RY* = *Y*/ *Y_m_*). Regression analyses using both linear and non-linear yield functions were tested, but the model selected was:

(1) $$RY=A+\frac{B}{1+D\times pH}$$

where *A* is a constant, such that *RY* tends towards *A* as the pH increases, while *B* and *D* model the curvature. Previous studies have also used expressions of Eq. (1) to model pH-yield relations (Liu et al., 2004; Slattery and Coventry, 1993).

The regression analysis included testing for the significance of the main plot treatments (Table S1). Thus, using Eq. (1) each crop type was tested for the effects according to four factors: site, year, P and K. Where a significant fertiliser P effect was detected the *RY* was calculated using a specific *Y_m_* according to the P treatments. In this case, *RY* was bifurcated according to added P levels (+P) and the P control (-P). After 1980 + P is equivalent to P1, P2 and P3 treatments; see Table 3 for further details on the P fertiliser treatments.

The fit of Eq. (1) was compared using a single equation for all levels of the treatment by allowing the parameters to vary; i.e. allowing both the linear parameters *A* and *B* to depend on the treatment; and allowing all parameters to depend on the treatment. The best fit was selected and the relevant metrics (*P* value, R^2^ value and parameter estimates with SE) were calculated accordingly. For each crop type with a significant yield-pH fit the predicted soil pH was determined at 90% *RY*. All statistical analyses were performed using GenStat 17 (VSN International, 2014).

## 3 Results

### 3.1 The effect of liming on soil pH, extractable P and exchangeable K

**Fig. 1:**
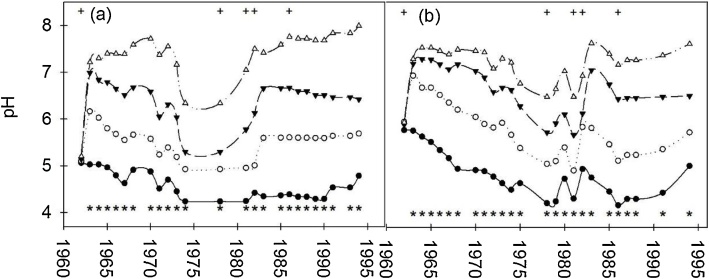
The effect of lime treatments on mean soil pH (a, b) over the course of the long-term liming experiment at Rothamsted and Woburn; four rates of lime were applied, the treatments were: control (⬤), low (○), medium (▾) and high (△). Rothamsted site: (a); Woburn site (b). * along the base of the *x* axis indicates a significant difference (*P* <  0.05) between the treatments; Along the top of (a) and (b) + marks the years in which lime was applied.

Liming treatments had a highly significant effect (*P* <  0.001) on increasing soil pH at both sites in every year of the experiment except for the first year (1962) when pH was measured before the lime was applied (Fig. 1a, d). The increases in soil pH immediately followed lime application, with decreases in pH where no lime was applied and when the effect of lime ended, i.e. when the lime had been used up. Lime additions were made six times over the course of the experiment (Table 2). The control treatment had the greatest decline in soil pH and this was more pronounced at the Woburn site. The soil pH values of the control treatment were mostly less than 5. In contrast the highest lime treatment had the largest increase in pH and had the least change after liming of all the treatments with pH values between 7 and 8. Correspondingly, the low and medium lime treatments had pH values which varied between pH 5 and 7. Whole plot treatment (lime, P and K fertiliser) effects and their interaction on soil pH are given for Rothamsted (Table S5) and Woburn (Table S6). At Rothamsted P fertiliser had a significant, but inconsistent effect on pH (*P* <  0.05) in 1983 and 1985, but there were no effects of K fertiliser nor any interactions between pH, P or K (for all combinations thereof) (Table S5). At Woburn there were four years (1968, 1970, 1973, 1981) where P fertiliser had a significant negative effect on pH, while K had a significant negative effect on pH in 1968 (Table S6).

Soil extractable P analysis (Olsen P) was undertaken for a selected number of years over the course of the liming experiment (Table 4). At Rothamsted in five of the years measured (1972, 1982, 1986, 1989, 1994) there was a significant, but inconsistent effect of liming on soil P. In Table 4 it is important to note the contrasting and different effects of liming according to the antecedent P level. For instance, in most of these years liming decreased the extractable P in the control P treatments, while in the treatments with added P (P1, P2, P3) liming increased the extractable P. Liming had no significant effect on extractable P in three years (1968, 1973, 1981) but a highly significant lime and P fertiliser effect was detected on extractable P at Woburn in six years (1973, 1981, 1982, 1986, 1989, 1994) (Table 4; Table S7). Overall at either site, there was no consistent increase or decrease in soil extractable P caused by liming. P fertiliser significantly (*P* <  0.001) increased soil extractable P in all years, but there was no lime ☓ P treatment interactions at Rothamsted (Table S7). At all four liming rates the control P treatment had the smallest extractable P value (Table 4). At Woburn the P fertiliser had the same effect as at Rothamsted and the P fertiliser significantly increased the extractable P (Table S7). There were three years (1986, 1989, 1994) out of six where there was a lime ☓ P fertiliser interaction detected (Table S7).

**Table 4:** The effect of lime and P fertiliser treatments on mean soil extractable P (Olsen) (ppm P in soil) over the course of the long-term liming experiment at Rothamsted and Woburn.

Year	Site	P treatment	Control (lime)	Low	Medium	High	SED
1968	Rothamsted	control P	15.35	13.6	16.15	18.15	1.56
1968	Rothamsted	+P	31.5	26.35	29.4	25.15	
1972	Rothamsted	control P	9.4	8.35	9.1	14.1	1.27
1972	Rothamsted	+P	29.65	24.45	25.35	29.55	
1973	Rothamsted	control P	8.3	7.5	8.45	12.45	1.10
1973	Rothamsted	+P	25.75	20.65	22.75	23.6	
1981	Rothamsted	control P	10.9	8.5	9	8.1	2.08
1981	Rothamsted	P1	10	8.6	11	10.7	
1981	Rothamsted	P2	25	23.5	24	30.2	
1981	Rothamsted	P3	30.6	29.95	27.9	30	
1982	Rothamsted	control P	11.9	9.1	8.1	6.5	1.64
1982	Rothamsted	P1	21.6	18.8	19	20.8	
1982	Rothamsted	P2	25.5	23	19.9	31.9	
1982	Rothamsted	P3	37.1	34.3	37.2	43.1	
1986	Rothamsted	control P	12.7	8.5	8.6	8.6	1.89
1986	Rothamsted	P1	12	7	10.1	11.5	
1986	Rothamsted	P2	26.1	19.1	24.4	34.2	
1986	Rothamsted	P3	31.5	24.5	30.8	30.1	
1989	Rothamsted	control P	11.3	6.5	7.5	8.9	1.97
1989	Rothamsted	P1	13.7	8.2	11.5	13.5	
1989	Rothamsted	P2	28	20.9	23.4	36.1	
1989	Rothamsted	P3	37.7	29	28.1	41.1	
1994	Rothamsted	control P	12.05	6.65	7.4	8.4	1.33
1994	Rothamsted	P1	10.85	7.3	9.35	9.55	
1994	Rothamsted	P2	18.8	14.3	16.95	21.85	
1994	Rothamsted	P3	22.7	16.7	19.1	24	
1973	Woburn	control P	18.95	16	17.7	23.25	0.83
1973	Woburn	+P	39.45	33.9	34.95	40.35	
1981	Woburn	control P	17.5	11.9	14.9	15.8	1.11
1981	Woburn	P1	18.8	15	15.5	16.5	
1981	Woburn	P2	41.6	31.6	30.4	39.4	
1981	Woburn	P3	43	35	34.3	39.8	
1982	Woburn	control P	16.3	13.1	18.9	13.2	1.39
1982	Woburn	P1	28	17.6	15.4	18.9	
1982	Woburn	P2	32	22.1	22.4	27.7	
1982	Woburn	P3	30.3	24.7	32.1	31	
1986	Woburn	control P	17.6	11	13.9	14.7	0.79
1986	Woburn	P1	23.8	15.1	16.1	18.7	
1986	Woburn	P2	34	23	27	34.9	
1986	Woburn	P3	42.4	30.7	29.8	37	
1989	Woburn	control P	21.6	13.4	16.9	17.8	1.04
1989	Woburn	P1	29.6	19.6	20.8	24.2	
1989	Woburn	P2	39.9	28.9	29.3	38.6	
1989	Woburn	P3	45.1	35.5	36.5	44.2	
1994	Woburn	control P	15.85	12.6	13.9	15.05	0.86
1994	Woburn	P1	24.3	14.35	14.4	17.4	
1994	Woburn	P2	27.85	18.9	20.5	28.1	
1994	Woburn	P3	29.05	22.8	24.35	30.6	

Soil exchangeable K was measured at Rothamsted and Woburn (Table S8). The only year at Rothamsted when a significant negative effect of liming on exchangeable K was observed was in 1972, while in 1964 there was a significant K fertiliser ☓ lime interaction (Table S9). At Woburn in three out of seven years there was a significant negative effect of liming on soil exchangeable K (Table S9). At both sites the control treatment always had the greatest exchangeable K values (Table S8). Applying K fertiliser significantly (*P* <  0.001) increased soil K for all years and in just one year (1967) there was a lime ☓ K fertiliser interaction (*P* = 0.022).

### 3.2 The effect of liming and P and K fertilisers on crop yield in the long-term liming experiment

Over the course of the liming experiment there was a total of 52 site/ crop years with yield data at the plot level. Analysis of the years when crop yields were recorded identified significant lime and P and K fertiliser effects at both sites (Table 5). The mean crop yields (t ha^−1^) for the liming treatments are given for Rothamsted (Table 6) and Woburn (Table 7). At each site the effect of lime significantly increased crop yield in most years. However, at Rothamsted there was no significant difference detected in 1964, 1968, 1974, 1977, 1982 and 1991, while at Woburn no effect on crop yield was found in 1968, 1981, 1982 and 1986. Thus, overall lime significantly increased yield (i.e. positive effect) for a wide range of crops tested. In several years (17 years at Rothamsted and 19 years at Woburn) there was a significant positive effect of P on yield, while positive effects of K on yield were only detected in four years at Rothamsted and in nine years in Woburn, out of a total of 14 possible years (Table 5).

**Table 5:** The significance level (*P* value)^a^ for the lime, P and K treatment effects for crop yield in each harvested year of the long-term liming experiment at Rothamsted and Woburn 1962–1996.

Harvest year	Crop	Rothamsted Lime	P	K^b^	Woburn Lime	P	K^b^
1963	Spring beans	<0.001	ns	<0.001	0.005	ns	<0.001
1964	Spring beans	ns	ns	0.028	<0.001	0.044	<0.001
1965	Spring barley	0.005	ns	ns	<0.001	<0.001	ns
1966	Spring barley	<0.001	ns	ns	<0.001	<0.001	ns
1967	Spring barley	<0.001	0.001	ns	0.004	<0.001	0.028
1968	Potatoes	ns	0.003	<0.001	ns	<0.001	<0.001
1970	Spring barley	<0.001	0.026	ns	<0.001	<0.001	0.003
1971	Spring barley	<0.001	0.004	ns	<0.001	<0.001	<0.001
1972	Spring barley	<0.001	0.005	ns	<0.001	0.006	<0.001
1973	Spring barley	<0.001	<0.001	ns	0.004	<0.001	ns
1974	Potatoes	ns	<0.001	<0.001	<0.001	<0.001	<0.001
1975	Spring oats	<0.001	<0.001	ns	<0.001	ns	ns
1977	Spring oats	ns	<0.001	ns	0.002	<0.001	ns
1978	Spring barley	<0.001	0.005	ns	<0.001	ns	0.002
1981	Spring oats	0.013	ns	–	ns	ns	–
1982	Spring oats	ns	0.004	–	ns	0.002	–
1983	Potatoes	<0.001	<0.001	–	<0.001	<0.001	–
1985	Spring barley	<0.001	<0.001	–	<0.001	0.002	–
1986	Winter Triticale	<0.001	<0.001	–	ns	0.049	–
1987	Spring lupins	<0.001	<0.001	–	0.016	0.003	–
1988	Linseed	<0.001	ns	–	<0.001	0.022	–
1989	Spring beans	<0.001	ns	–	<0.001	ns	–
1990^c^	Spring beans	<0.001	0.017	–	–	–	–
1991	Winter OSR	ns	ns	–	<0.001	0.011	–
1993^c^	Winter lupins	<0.001	ns	–	–	–	–
1995	Winter wheat	<0.001	0.041	–	<0.001	0.028	–
1996	Winter wheat	<0.001	ns	–	<0.001	ns	–

a ns indicates a *P* value > 0.05.

b From 1981 onwards there was no K main plot treatment.

c Crop failure at Woburn only.

**Table 6:** The mean crop yield^a^ (t ha^−1^) for the four liming treatments (control, Low, Medium and High) at Rothamsted 1962–1996.

Harvest year	Crop	Control	Low	Medium	High	SED
1962^b^	Spring beans	1.54	2.01	2.55	2.33	–
1963	Spring beans	1.34	2.59	2.89	2.82	0.458
1964	Spring beans	1.85	2.38	2.48	2.15	0.223
1965	Spring barley	3.25	5.25	5.24	5.18	0.545
1966	Spring barley	2.73	4.41	4.77	4.80	0.411
1967	Spring barley	1.44	4.33	4.22	3.87	0.449
1968	Potatoes	23.07	26.07	26.82	24.95	2.488
1969	Fallow	–	–	–	–	–
1970	Spring barley	0.31^d^	2.87	3.64	3.58	0.265
1971	Spring barley	0.67^d^	3.54	4.34	4.53	0.406
1972	Spring barley	0.00^c^	3.67	4.55	4.90	0.484
1973	Spring barley	0.00^c^	3.24	4.07	4.57	0.394
1974	Potatoes	23.2	31.7	34.2	34.3	4.182
1975	Spring oats	1.85	2.42	2.86	2.80	0.204
1976^c^	Spring oilseed rape	–	–	–	–	–
1977	Spring oats	3.26	3.47	3.77	3.58	0.232
1978	Spring barley	0.19^d^	2.33^e^	4.01	4.20	0.502
1979	Fallow	–	–	–	–	–
1980	Fallow	–	–	–	–	–
1981	Spring oats	3.34	3.56	3.54	3.08	0.139
1982	Spring oats	1.42	1.48	1.31	1.38	0.090
1983	Potatoes	23.83	29.51	30.03	28.84	0.994
1984	Fallow	–	–	–	–	–
1985	Spring barley	0.00^c^	6.07	7.51	7.77	0.356
1986	Winter Triticale	6.24	8.00	8.27	8.20	0.245
1987	Spring lupins	1.82	2.80	2.87	3.10	0.233
1988	Linseed	0.00^c^	2.69	2.77	2.66	0.118
1989	Spring beans	0.06^d^	0.90	1.04	1.40	0.185
1990	Spring beans	0.12^d^	1.91	2.57	3.04	0.178
1991	Winter oilseed rape	1.39	2.38	2.12	2.56	0.894
1992^c^	Winter oilseed rape	–	–	–	–	–
1993	Winter lupins	0.38^d^	2.19	1.61	1.41	0.2200
1994^c^	Winter lupins	–	–	–	–	–
1995	Winter wheat	0.73^d^	6.81	7.76	7.84	0.492
1996	Winter wheat	2.74^d^	8.30	8.79	8.63	0.846

a All grain yield (including lupins) has been standardised to 85% dry matter, oilseeds to 90% dry matter and potato yield is fresh weight.

b Treatment mean data only, no plot level data available.

c Crop failure.

d Some plots failed for this treatment.

**Table 7:** The mean crop yield^a^ (t ha^−1^) for the four liming treatments (control, Low, Medium, High) at Woburn 1962–1996.

Harvest year	Crop	Control	Low	Medium	High	SED
1962^b^	Spring beans	1.86	2.38	2.40	2.76	–
1963	Spring beans	1.56	2.20	2.07	2.07	0.148
1964	Spring beans	2.40	2.07	1.63	1.66	0.102
1965	Spring barley	4.77	4.99	5.29	5.32	0.060
1966	Spring barley	4.63	4.96	5.15	5.14	0.080
1967	Spring barley	3.64	4.20	4.36	4.40	0.191
1968	Potatoes	26.79	25.88	24.18	24.50	1.282
1969	Fallow	–	–	–	–	–
1970	Spring barley	1.52	3.77	4.10	4.24	0.131
1971	Spring barley	2.18^d^	4.13	4.19	4.24	0.141
1972	Spring barley	5.31^d^	4.81	5.28	5.83	0.186
1973	Spring barley	4.19^d^	3.67	4.17	4.73	0.239
1974	Potatoes	17.9	25.2	26.8	27.8	1.930
1975	Spring oats	1.51	2.07	2.11	2.17	0.091
1976^c^	Spring oilseed rape	–	–	–	–	–
1977	Spring oats	2.44	2.63	2.67	2.91	0.095
1978	Spring barley	1.21^d^	4.22	4.82	5.03	0.183
1979	Fallow	–	–	–	–	–
1980	Fallow	–	–	–	–	–
1981	Spring oats	3.92	3.80	3.70	3.60	0.179
1982	Spring oats	1.64	1.85	1.83	1.84	0.162
1983	Potatoes	39.6	48.1	41.2	39.0	1.606
1984	Fallow	–	–	–	–	–
1985	Spring barley	0.78	6.40	7.45	7.45	0.213
1986	Winter Triticale	6.76	6.73	6.55	6.71	0.549
1987	Spring lupins	1.96	1.71	1.61	1.62	0.410
1988	Linseed	1.31	2.76	2.77	2.47	0.116
1989	Spring beans	0.18^d^	0.61	1.00	1.30	0.295
1990^c^	Spring beans	–	–	–	–	–
1991	Winter oilseed rape	1.16	2.42	2.62	2.69	0.473
1992^c^	Winter oilseed rape	–	–	–	–	–
1993^c^	Winter lupins	–	–	–	–	–
1994^c^	Winter lupins	–	–	–	–	–
1995	Winter wheat	1.39	7.78	7.37	7.33	1.480
1996	Winter wheat	3.85	8.10	7.48	7.56	1.549

a All grain yield (including lupins) has been standardised to 85% dry matter, oilseeds to 90% dry matter and potato yield is fresh weight.

b Treatment mean data only, no plot level data available.

c Crop failure.

d Some plots failed for this treatment.

Looking at specific crops in more detail: The crop grown most frequently, in nine years of the experiment, was spring barley and lime had a significant positive yield effect in all years (Table 5). At both sites the yield of spring barley in 1985 from the liming treatments was much greater than the other eight years; overall there were four different spring barley varieties grown (Table 1). In addition, the P fertiliser significantly increased yield in most years, except for two years (1965, 1966) at Rothamsted and one year (1978) at Woburn. In comparison, there were no K fertiliser effects at Rothamsted, but four years where K significantly increased yield at Woburn. For spring beans there were significant positive effects of lime on yield at Rothamsted in three out of four years and also in all three crop years at Woburn (Table 5). Of the three years with potatoes liming only had a significant positive effect on yield in a single year (1983) at Rothamsted, but in two years (1974 and 1983) at Woburn. The P fertiliser treatment effects were positive and were detected for potato yield in all years at both sites; also, K fertiliser increased potato yield significantly at both sites in 1968 and 1974 (Table 5). For winter triticale (1986) there were significant positive effects of lime on yield only at Rothamsted. A positive lime effect was detected on the yield of winter lupins (1993), while at Woburn the winter lupin crop failed. Some crops varied in their response between years. For example, spring oats (at Rothamsted) responded to lime in 1975 and 1981, but there was no effect on yield in 1977 or 1982. In comparison, spring oats at Woburn showed positive effects of liming in 1975 and 1977, but not in 1981 or 1982. In 1991 the winter oilseed rape yield was significantly positively increased from liming at Woburn, but not at Rothamsted (Table 5). In 1995 and 1996 lime significantly increased the yield of winter wheat at both sites (Table 5). Treatment interactions of crop yield (Table S10) show the complexity of the data and provide clear evidence of differences between sites. Overall, for several crops (in multiple years) there were large differences between the sites in terms of responsiveness of crop yield to lime (Table 5). Moreover, the importance of these differences is demonstrated below in the soil pH-yield relationship for selected crops (Fig. 3).

### 3.3 Year effects on the relationship between crop yield and soil pH

Over the course of the experiment there were large contrasts between years in climate as shown in the data for precipitation (i.e. rainfall), cumulative temperature and solar radiation during the growing seasons in Supplementary Tables S2, S3 and S4. Because the experiment included both winter and spring crops it is most useful to consider climate variables for the growing season (April to July) only. The mean growing season rainfall was 210 mm at Rothamsted and 207 mm at Woburn. In the driest year (1976) there was < 90 mm at both sites and crops failed due to drought. In the wetter years there was >250 mm rainfall, but no observations of waterlogging or crop failure. Rainfall clearly had a major effect on crop yield in each year of the experiment. Analysis of long-term data of winter wheat yield and climate showed that 33% of variability in grain yield was explained by rainfall and temperature (Chmielewski and Potts, 1995). In the LTL experiment, yields of spring barley had a weak positive relationship with growing season rainfall (data not shown). Cumulative temperature records show very little difference between the sites. At Rothamsted the mean growing season cumulative temperature (>0 °C) was 2055 °C, while it was 2085 °C at Woburn. At each site large inter-year variability in solar radiation was observed and, in combination with the other environmental factors, solar radiation explains the potential range for crops to produce dry matter (Monteith and Moss, 1977).

**Fig. 2:**
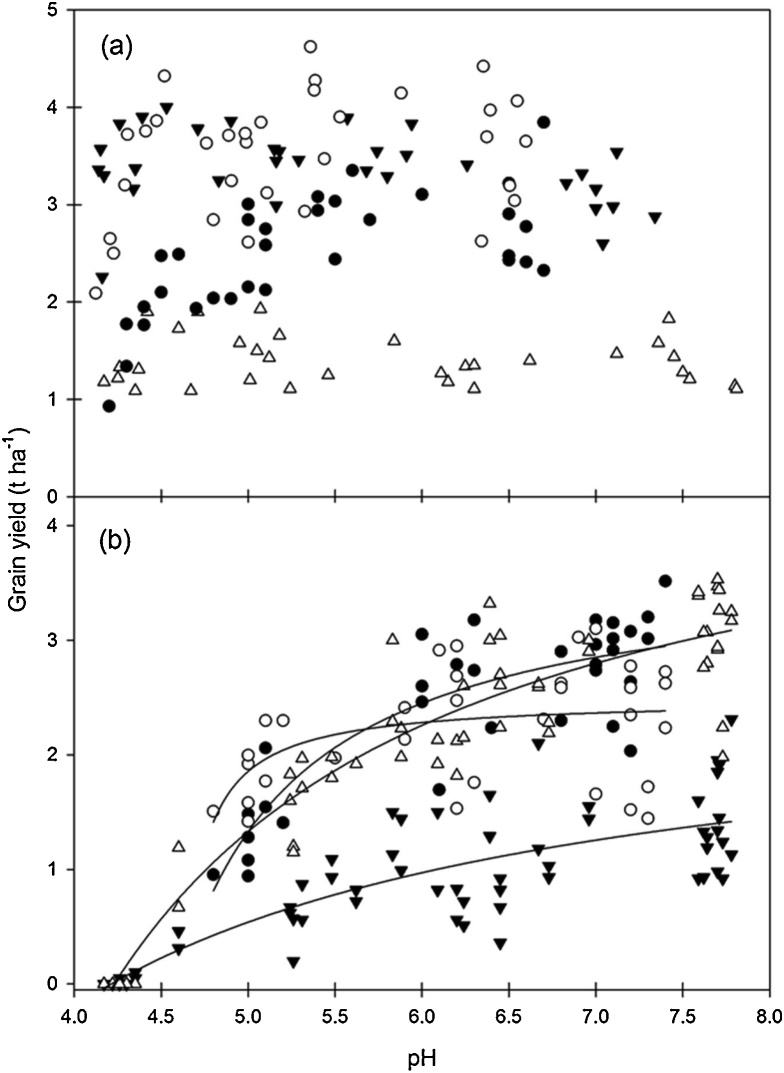
The relationship between grain yield (t ha^−1^) and soil pH for (a) spring oats in 1975 (⬤), 1977 (○), 1981 (▾), 1982 (△) and (b) spring beans in 1963 (⬤), 1964 (○), 1989 (▾) and 1990 (△) at Rothamsted; in (b) the regression curves represent significantly different fits for separate years for spring beans.

An analysis of the soil pH-yield relationship using data that combined all years found that ‘year’ was always a highly significant (*P* <  0.001) factor. To illustrate the importance of year, the yield-pH data for spring oats is given for 1975, 1977, 1981 and 1982 at Rothamsted (Fig. 2a). Yields were much higher in 1977 and 1981 than in 1975 and 1982. This could be for a range of different reasons. It was probably not due to temperature or solar radiation as there were no large differences for these years, but there was much less rainfall in 1975 and 1977 compared with 1981 and 1982 (Table S2, S3, S4). In addition to climate there are numerous other biotic and management (agronomic) factors which could explain differences in crop yield. There was very similar agronomic management (e.g. crop inputs) between the 1981 and 1982 spring oat crops, including the same amount of basal N fertiliser applied. The significantly greater crop yield in 1981 could be related to the longer growing period (over two weeks more) than for 1982. Moreover, the 1981 crop was preceded by two fallow years and this may have provided a significant additional benefit towards the final yield. Indeed, Mann (1943) described a one- or two-year fallow as providing beneficial effects. Overall for spring oats there was no significant relationship between crop yield and soil pH at Rothamsted (Fig. 2a) or Woburn (data not shown).

**Fig. 3:**
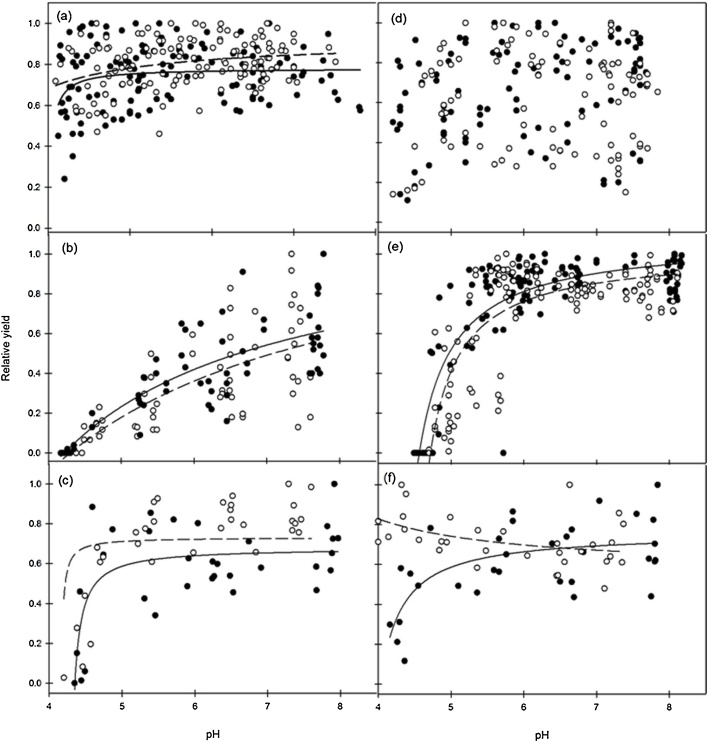
Relationship between crop relative yield (*RY*) and pH only for spring oats (a), spring beans (1989 data only) (b), winter oilseed rape (c), potato (d), winter wheat (e) and spring lupins (f) at the Rothamsted (⬤) and Woburn (○) sites; regression fit for Rothamsted are given with solid lines and for Woburn with dashed lines. For actual crop yield (t ha^−1^) refer to Table 6, Table 7.

The significant effect of year on yield is also illustrated by data on spring beans at Rothamsted (Fig. 2b). Here there was a significant yield-pH relationship, but the nature of the relationship differed in each of the four years. These differences could be explained by a variety of factors, including climate and the use of different crop varieties. As a consequence of the year-to-year differences in yield it was decided to evaluate the yield-pH relationship using *RY* to standardise the data for a particular site or site/ treatment combination. *RY* is used subsequently to investigate the site and fertiliser P effects.

### 3.4 Site effects on the relationship between relative yield (RY) and soil pH

Site was found to have a significant influence on crop *RY*-pH relationships for all crops except for potato and as the winter lupin crop failed at Woburn no site comparison can be made. To illustrate the importance of site, six crops from the long-term experiment are presented as examples (Fig. 3). For spring oats (Fig. 3a) there was a significant difference between the sites for the *RY*-pH relationship. However, while the *RY*-pH function (Eq. 1) fitted the data there was a small coefficient of determination (R^2^ = 0.1) (Table 8). For potatoes there was no difference between sites for *RY* and there was also a weak fit (R^2^ = 0.059) for the *RY*-pH relationship (Fig. 3d; Table 8). Analysis of one year (1989) found no significant difference between sites for the *RY* of spring beans (Fig. 3b, Table 8), but for all years with spring beans there was a significant site effect (Table 8).

**Table 8:** Regression statistics (for all sites) and parameter coefficients (for individual sites) for the relationship between relative yield (*RY*) and soil pH described by Eq. 1 for different arable crops from the long-term liming experiment at Rothamsted and Woburn 1962–1996.

Crop	*P* value	R^2^	Site	A (± SE)	B (± SE)	D (± SE)
Spring oats	<0.001	0.10	Rothamsted	0.78 (0.02)	0.007 (0.008)	−0.25 (0.01)
			Woburn	0.92 (0.14)	0.128 (0.43)	−0.38 (0.42)
			(Sign. level)	**	ns	ns
Potato	0.006	0.059	Rothamsted	0.79 (0.13)	0.09 (0.21)	−0.31 (0.149)
			Woburn	0.67 (0.04)	0.01 (0.02)	−0.25 (0.007)
			(Sign. level)	ns	ns	ns
Spring Beans^a^	<0.001	0.603	Rothamsted	1.139 (0.368)	3.01 (7.83)	−0.87 (1.44)
			Woburn	1.44 (1.01)	−7.6 (32.1)	1.01 (6.07)
			(Sign. level)	ns	ns	ns
Spring Beans^b^	<0.001	0.592	Rothamsted	1.063 (0.09)	0.50 (0.24)	−0.35 (0.04)
			Woburn	0.897 (1.22)	0.392 (0.272)	−0.33 (0.059)
			(Sign. level)	***	ns	ns
Winter wheat	<0.001	0.723	Rothamsted	1.076 (0.043)	0.123 (0.028)	−0.244 (0.005)
			Woburn	1.008 (0.046)	0.097 (0.027)	−0.232 (0.005)
			(Sign. level)	***	*	ns
Winter oilseed rape	<0.001	0.619	Rothamsted	0.680 (0.042)	0.0165 (0.00973)	−0.235 (0.0036)
			Woburn	0.986 (0.076)	0.1065 (0.052)	−0.263 (0.0128)
			(Sign. level)	***	ns	*
Spring lupins	<0.001	0.377	Rothamsted	0.761 (0.071)	0.058 (0.056)	−0.267 (0.026)
			Woburn	0.568 (0.3)	−0.26 (1.74)	−0.5 (1.44)
			(Sign. level)	**	***	ns

*The parameter coefficients are significantly different between sites at P < 0.05.

**The parameter coefficients are significantly different between sites at P < 0.01.

***The parameter coefficients are significantly different between sites at P < 0.001.

a These values represent regression analysis for 1989 data only and correspond with data shown in Fig. 3b.

b These values represent regression analysis for all years of spring beans data; see Table 1 for further details.

There were significant effects of site on the *RY* of winter oilseed rape (Fig. 3c), winter wheat (Fig. 3e), and spring lupins (Fig. 3f). The differences between the *RY*-pH relationship for each crop are shown in the parameter coefficients (Table 8). For these three crops Eq. (1) fitted the data well and a high coefficient of determination (R^2^) was calculated for winter wheat (0.72), while it was lower for winter oilseed rape (0.62) and spring lupins (0.38). The *RY* of winter oilseed rape was more responsive to pH at Woburn than at Rothamsted (Fig. 3c; Table 8), although the large variability in *RY* meant that it was not possible to predict the soil pH at 90% *RY* accurately. At both sites the winter wheat *RY* was consistently responsive to soil pH and the two years of data provide a satisfactory range of *RY* values across a wide pH spectrum (Fig. 3e). Previous studies (Liu et al., 2004; Slattery and Coventry, 1993) have also determined a *RY*-pH relationship using the same model for wheat. The response of wheat at Rothamsted was stronger than that at Woburn and the model (Eq. 1) was significantly different between the sites (Table 8). For spring lupins there was a significant effect of site on the *RY*-pH relationship, but only the *RY* at Rothamsted responded to pH. For spring lupins at Woburn there was no pH response and a weak fit to Eq. 1 (Table 8).

These site effects reflect differences between the climate and soil properties at Rothamsted and Woburn (see *Experimental site description* above). However, since the differences in climate were small (Tables S2, S3, S4) the differences are most likely to be due to soil properties, especially (*i*) greater clay content at Rothamsted than at Woburn, and (*ii*) the greater water holding capacity of the Rothamsted soil than the Woburn soil.

### 3.5 Effects of P fertiliser on the relationship between crop relative yield (RY)and soil pH

**Fig. 4:**
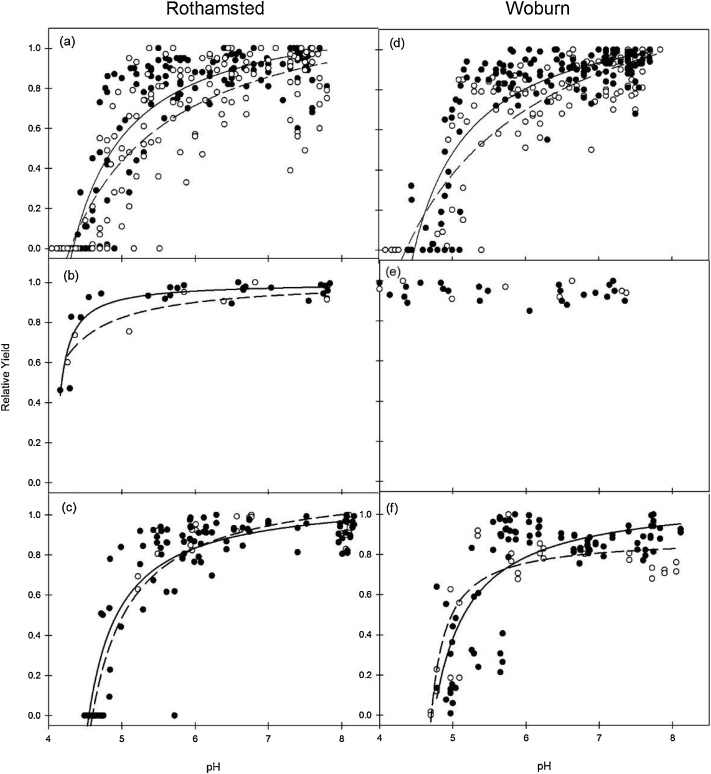
Relationship between crop relative yield (*RY*) and pH with the effect of phosphorus (⬤ +P, ○ -P) for spring barley (a, d), winter triticale (b, e) and winter wheat (c, f) at the Rothamsted (a, b, c) and Woburn (d, e, f) sites; regression fit for + P are given with solid lines, for -P with dashed lines and a single dotted line where there was no difference between + P and -P. For actual crop yield (t ha^−1^) refer to Table 6, Table 7.

For some crop types there was a significant positive effect of P fertiliser on the *RY*-pH relationship. Fig. 4 illustrates this at each site for spring barley, winter triticale and winter wheat. Spring barley had the most measurement years of any crop and provides the most powerful *RY*-pH data for this whole experiment. At both sites the *RY* of spring barley was clearly responsive to pH (Fig. 4a, d) and there were significant positive P effects as well (Table 9). At both sites the *RY*-pH relationship was more responsive for + P than for –P and significant differences were detected by Eq. 1, e.g. parameter A and D were significantly different (Table 9). The *RY* of winter triticale at Rothamsted was responsive to pH and there was a highly significant P effect for the model of the *RY*-pH relationship (Fig. 4 b, Table 9). In contrast at Woburn the model did not fit significantly and thus no pH response or P effect was detected (Fig. 4e, Table 9). For winter wheat at both sites there was a significant positive P effect on the *RY*-pH relationship (Fig. 4c, f; Table 9). There was also a P effect for the winter wheat at Woburn and the model fit for the + P was significantly greater than for the –P treatment with a difference in the B parameter (Table 9). In addition to the examples given in Fig. 4, other crops were investigated for a P effect on the *RY*-pH relationship. No P effect was detected for linseed or spring beans, but there was for winter oilseed rape (Table 9).

**Table 9:** Regression statistics (for all sites) and parameter coefficients (for the phosphorus effect: with + P^a^; without -P) for the relationship between relative yield (*RY*) and soil pH described by Eq. 1 for several different arable crops from the long-term liming experiment at Rothamsted and Woburn 1962–1996.

Crop	Site	Fert. P level	*P* value	R^2^	A (± SE)	B (± SE)	D (± SE)
Spring Barley	Rothamsted	+P	<0.001	0.698	1.228 (0.062)	0.287 (0.071)	−0.285 (0.012)
Spring Barley	Rothamsted	-P			1.297 (0.121)	0.622 (0.272)	−0.344 (0.041)
		(Sign. effect)			***	ns	*
Spring Barley	Woburn	+P	<0.001	0.707	1.236 (0.064)	0.276 (0.076)	−0.273 (0.013)
Spring Barley	Woburn	-P			1.542 (0.156)	1.271 (0.672)	−0.418 (0.084)
		(Sign. effect)			**	ns	***
Winter Triticale	Rothamsted	+P	<0.001	0.81	0.999 (0.022)	0.021 (0.007)	−0.249 (0.003)
Winter Triticale	Rothamsted	-P			0.95 (0.105)	0.075 (0.127)	−0.284 (0.074)
		(Sign. effect)			***	ns	ns
Winter Triticale	Woburn	+P/ -P	ns	b	–	–	–
Winter Wheat	Rothamsted	+P	<0.001	0.819	1.093 (0.047)	0.119 (0.029)	−0.244 (0.005)
Winter Wheat	Rothamsted	-P			1.027 (0.086)	0.142 (0.065)	−0.248 (0.012)
		(Sign. effect)			**	ns	ns
Winter Wheat	Woburn	+P	<0.001	0.594	1.059 (0.07)	0.121 (0.049)	−0.235 (0.011)
	Woburn	-P			0.883 (0.064)	0.042 (0.022)	−0.222 (0.005)
		(Sign. effect)			ns	*	ns
Linseed	Rothamsted	+P	<0.001	0.845	1.21 (0.154)	0.369 (0.232)	−0.303 (0.036)
	Rothamsted	-P			0.973 (0.16)	0.11 (0.118)	−0.259 (0.0247)
		(Sign. level)			ns	ns	ns
Linseed	Woburn	+P	<0.001	0.594	0.978 (0.091)	0.090 (0.081)	−0.286 (0.032)
Linseed	Woburn	-P			0.940 (0.141)	0.098 (0.112)	−0.280 (0.032)
		(Sign. level)			ns	ns	ns
Winter Oilseed rape	Rothamsted	+P	<0.001	0.415	0.747 (0.054)	0.009 (0.008)	−0.233 (0.002)
	Rothamsted	-P			1.19 (0.733)	0.89 (3.05)	−0.411 (0.52)
		(Sign. effect)			ns	ns	*
Winter Oilseed rape	Woburn	+P	<0.001	0.763	0.93 (0.053)	0.046 (0.023)	−0.242 (0.007)
	Woburn	-P			1.08 (0.165)	0.275 (0.228)	−0.298 (0.044)
		(Sign. effect)			ns	ns	*

*The parameter coefficients are significantly different between the added P levels at P < 0.05.

**The parameter coefficients are significantly different between the added P levels at P < 0.01.

***The parameter coefficients are significantly different between the added P levels at P < 0.001.

a After 1980 + P is equivalent to P1, P2 and P3 treatments; see Table 3 for further details on the P treatments.

b Residual variance exceeds variance of response variate.

## 4 Discussion

### 4.1 Evaluation of the impact of liming on soil pH, extractable P and exchangeable K

The significant (*P* <  0.001) increases in soil pH data Fig. 1a, b) after lime was applied are consistent with expectations for these treatments. Indeed, pH decreased (i.e. there was soil acidification) most for the control and low lime treatments, while the high lime treatment had the greatest pH increase. A small difference was observed in the general nature of pH changes between the sites, with the Woburn site slightly more responsive. These differences reflect the soil types at each site with the greater sand content of the Woburn soil corresponding with stronger acidification than the Rothamsted soil. A small increase in pH of the control treatment at both sites was observed towards the end of the experiment (Fig. 1a, b) and this is consistent with increases in pH due to recent reductions in atmospheric S deposition across Great Britain (Reynolds et al., 2013). The soil pH data (Fig. 1a, b) were used to develop the RothLime model (http://www.rothamsted.ac.uk/rothlime; (Goulding et al., 1989). RothLime provides useful recommendations for farmers and managers, a very practical and valuable outcome from the LTL experiment. Subsequent analysis of the soil pH after the experiment had finished showed that the changes in soil pH significantly affected the rate of soil C and N cycling (Kemmitt et al., 2006) and, in raising the pH, the liming treatments increased soil microbial activity.

Considering the results from both sites, in selected years liming did increase P availability as measured by the Olsen method (Table 4; Table S7). Likewise, Simonsson et al. (2018) recently showed that liming increased soil P availability in long-term experiments in Sweden, but they determined P availability using an ammonium lactate extractant. It is interesting to note the effects of both liming and added (fertiliser) P on extractable P at Rothamsted and Woburn (Table 4). The wide range of extractable P values is not surprising since Johnston et al. (2013) also reported a wide range of critical Olsen P values for arable crops with similar soil types to those of this study. Indeed, at both sites the P effect was complex and there was large variability in the extractable P responses observed (i.e. increasing/ decreasing or positive/ negative effects). Furthermore, the importance of Olsen P for crop yield is strongly related to other soil conditions such as soil organic matter, soil N and soil structure (Poulton et al., 2013).

The negative effects (i.e. decreasing availability) of liming on exchangeable K (Table S8; Table S9) are consistent with previous studies on the kinetics of K release for these soils (Goulding, 1981). Analysis of K dynamics in the Rothamsted and Woburn soils has found that the release of K is directly related to the percentage of clay (Addiscott and Johnston, 1975). Therefore, because Rothamsted soil has greater clay (21 vs 11%) than Woburn it is to be expected that the exchangeable K would be greater in the Rothamsted soil, than the Woburn soil (Table S8). The different responses to pH for the soils at each site are largely a function of the soil texture. It is suggested that the effect of lime to decrease the exchangeable K is also due to the added Ca (from the lime) which would displace K from cation exchange sites. In addition, the associated increased crop yield would increase the removal of K from the soil. Overall, there were a greater number of sub-plot (P and K) treatment effects and interactions with pH for the sandier Woburn soil (Table S6) than for the Rothamsted soil (Table S5). Further research is required to understand better the effect of liming on key soil properties such as P and K. For instance, the dynamic nature of liming on soil fertility in the LTL experiment is shown, but more detail of these significant effects is required.

### 4.2 Evaluation of crop yield response to soil pH

The crop yields in the LTL experiment (Table 6, Table 7) are much lower than are currently observed, e.g. from 2012 to 2016 mean UK barley yields were 6.1 t ha^−1^ and mean UK potato yields were 39.1 t ha^−1^ (FAO, 2018). A comparison between the crop yields at Rothamsted and Woburn in this LTL experiment with UK historic commercial yields (FAO, 2018) indicates that in general the yields were within a similar range to those from the same time period. There are many environmental factors which could explain differences in crop yield, also crop improvement via new varieties is an important factor. Such a comparison with current crop yield production does not diminish from the valuable insights the LTL experiment provides on the effect of pH on crop yield. Evaluation of the yield and *RY*-pH relationships (Fig. 2, Fig. 3, Fig. 4) shows the large differences in response between crops. In particular, two crops (oats and potato) stand out because they exhibited weak *RY*-pH relationships (Fig. 3a, d). This is generally consistent with previous studies e.g. Maier et al. (2002). However, potato tuber quality is also an issue. In the UK, low soil pH is recommended to control potato common scab (*Streptomyces* spp.) (AHDB, 2013), although this practice is not always effective with all *Streptomyces* spp. (Dees and Wanner, 2012). The potato *RY* data (Fig. 3d) from 1968, 1974 and 1983 did not provide any details on the presence of common scab. Thus, without quality data it was not possible to assess the full impact of liming on potato production. Furthermore, the potato yields varied across a wide range between sites and years (Table 6, Table 7). At Woburn there was a highly significant positive effect of liming on yield in 1974 and 1983, while at Rothamsted there was only an effect of liming on yield in 1983 (Table 5). Such between-year and site differences make it difficult to provide a consistent or clear indication of the *RY*-pH relationship for potato.

For oats there was a very weak *RY*-pH relationship (Fig. 3a) and there was a significant difference in yield between years (e.g. 1981 and 1982). Oat varieties have a range of tolerance to aluminium (Al^3+^) (Foy et al., 1987; Nava et al., 2006) but are thought to cope with acidic soil better than other cereal crops. Some studies have reported responses in the yield of oats to lime (Li et al., 2001), but these are unusual. The very significant year effect on the *RY*-pH relationship of oats is intriguing (Fig. 2a) and raises questions about why this occurred.

Cereal crops other than oats showed positive yield responses to liming. Significant site and P fertiliser effects were observed for the spring barley *RY*-pH relationship (Fig. 4a, d; Table 9). Several previous studies have also reported that increased yields resulting from liming are associated with increased pH (Dolling et al., 1991a; Farhoodi and Coventry, 2008; Liu et al., 2004; Slattery and Coventry, 1993). In some previous research liming has been described as alleviating Al^3+^ toxicity (Dolling et al., 1991a). Indeed Foy (1988) reported distinct differences in Al^3+^ tolerance (and hence sensitivity) between plants which is characteristic of their natural genetic variation. Also, analysis of soil samples from both sites, taken three years after the LTL experiment finished showed very large differences in exchangeable Al^3+^ between the liming treatments (Kemmitt et al., 2006). However, because exchangeable Al^3+^ was not measured during the LTL experiment no comment can be made on this, although it is likely that exchangeable Al^3+^ was only at excessive levels in soil at the lowest pH values (i.e. < pH 4.3). The importance of P status on yield response to pH has recently been reported for barley in Germany (von Tucher et al., 2018) and Ethiopia (Alemu et al., 2017). Von Tucher et al (2018) concluded that for barley (and wheat) liming soils with low pH increases fertiliser use efficiency. In this study a lack of P (i.e. -P; P control treatment) resulted in a significantly reduced yield response for barley (Fig. 4a, d), triticale (Fig. 4b, e) and wheat (Fig. 4c, f). A significant effect of P fertiliser was also detected for winter oilseed rape (Table 8). Differences also exist between varieties of the same crop type. Some varieties of winter wheat have greater Al^3+^ tolerance and hence do not respond to lime (Dolling et al., 1991b). A striking example of the difference in crop response to liming in this study is when and where crops failed. For example, in 1985 at Rothamsted spring barley growing on plots with pH 4.0 failed (Table 6). In the following year, the triticale grown on the same plots gave yields of 5.5 t ha^−1^ (Table 6) and 6.5 t ha^−1^ at Woburn (Table 7). When compared with other cereal crops triticale has often shown to be more tolerant of soil acidity. In a study of the *RY*-pH relationship for wheat, barley and triticale, Liu et al. (2004) found that triticale was the least sensitive crop to pH. The *RY*-pH response curves are also much weaker for triticale than for barley (Slattery and Coventry, 1993). The *RY*-pH relationship is unique for each cereal crop type. The greatest and most consistent P-dependent lime response was for spring barley, followed by winter wheat and winter triticale was the least responsive to pH (Fig. 4).

Overall, there was a significant site effect for the *RY*-pH relationship for spring beans (Table 8). Spring beans showed large year differences at Rothamsted (Fig. 2b) and when only 1989 was considered there was no site effect (Fig. 3b; Table 6). There was no evidence of a P fertiliser effect on spring beans and there was large variability in *RY*. Nevertheless, increasing the soil pH through liming has direct benefits. Low soil pH has a negative effect on the ability of common beans (*P. vulgaris*) to nodulate (Frey and Blum, 1994). Similarly for lupins, Denton et al. (2017) found reduced nodulation at low soil pH. There are, though large differences in performance among lupin varieties. Some are sensitive to acidic soils while others to highly alkaline soils. Kerley et al. (2004) reported satisfactory shoot biomass production by lupins between soil pH 4.9 and 7.2. In the LTL experiment, significant site effects were found with a good yield response to lime for spring lupins at Rothamsted, but not at Woburn (Fig. 3f). For winter lupins there was a significant *RY*-pH relationship at Rothamsted, but the crop at Woburn failed (Table 7). Additional research is required to characterise the yield-pH relationship better for both beans and lupins.

There was a significant yield-pH relationship for winter oilseed rape at both sites which indicates a positive response to lime (Fig. 3c). Nevertheless, the relationship was weaker in comparison with that for winter wheat at Rothamsted, but correspondingly stronger at Woburn. This smaller yield response was also observed in a study comparing canola (i.e. same crop as oilseed rape) and three different cereal crops (Slattery and Coventry, 1993). This suggests that winter oilseed rape is more sensitive to acidic soils and might not tolerate Al^3+^ well. Lofton et al. (2010) showed that both extractable Al^3+^ and pH were related to the yield of winter canola. Furthermore, Lofton et al. (2010) reported that there was a difference in the response to pH between canola varieties.

Linseed is a minor crop and there have been very few studies on the effects of pH on linseed yield. Significant site effects were detected on the *RY*-pH relationship for linseed (Table 8). No P fertiliser effects were found at either site (Table 9). The linseed was significantly more sensitive to acidic soil compared with the spring lupins. At Rothamsted the control plots for the spring lupins had a *RY* of 0.71 in 1987, but in 1988 the same plots did not produce any yield for linseed (Table 6). Because of the significant seasonal effect on the yield-pH relationship there is a need for a greater number of years of data to understand the lime crop response better for winter oilseed rape and linseed.

### 4.3 Implications for future liming management

**Fig. 5:**
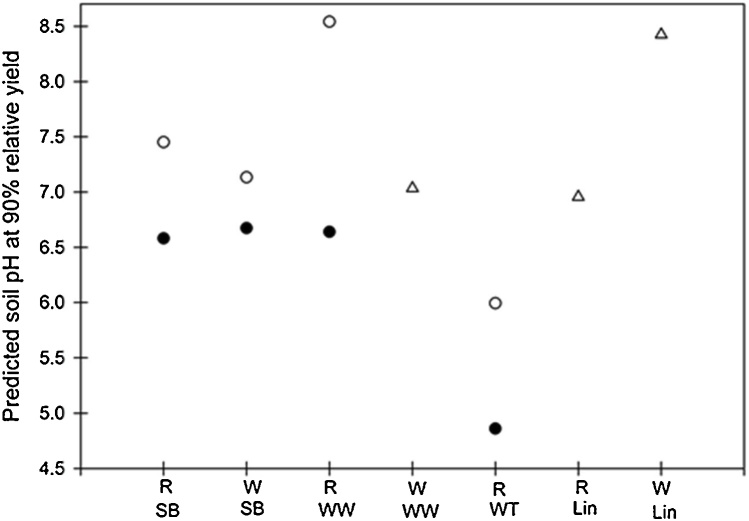
The critical soil pH at 90% relative yield for selected crops at the Rothamsted and Woburn sites. Rothamsted = R, Woburn = W, Spring barley = SB, Winter wheat = WW, Winter triticale = WT and Linseed = Lin; where there is a significant P effect a separate symbol is given for each crop: +P (⬤), -P (○), for crops with no P effect (△).

The *RY*-pH relationship (Fig. 3, Fig. 4) can be used to determine the critical pH at 90% *RY*. Calculation of the predicted pH at 90% *RY* is given for five crops (Fig. 5); with site and P effects shown when they were detected. For several crops (winter oilseed rape, spring beans, spring lupins) there was large variability in *RY* and it was not possible to predict the pH at 90% *RY*. The greatest site differences in critical pH were observed for winter wheat and linseed. For each crop the critical pH at Woburn was much greater than at Rothamsted: the critical pH for winter wheat on the sandier Woburn soil was 7.5 compared to 6.6 (+P) or 8.5 (-P) at Rothamsted and for linseed the critical pH was 8.4 compared to 7.0. This range indicates that soil type (i.e. site) can make a major difference to setting the critical soil pH. In contrast, for spring barley there were much smaller differences in critical pH and the only difference was for the critical pH without P (-P). The critical pH for spring barley (both sites), winter triticale (Rothamsted) and winter wheat (Rothamsted) without P (-P) was much greater than when P was added (+P) (Fig. 5). In comparison, the P level had no difference for winter wheat at Woburn nor for linseed at either site. These differences in critical pH indicate that where P inputs are reduced, then the critical pH increases and there is a greater need for liming. There is clearly a strong interaction between soil pH and P availability (Simonsson et al., 2018), which influences how P nutrition for arable crops is optimised. Barrow (2017) suggests that there is a need to re-evaluate the optimum soil pH for P uptake. However, pH is not the only soil property of importance: organic matter content also controls yield response to P (Johnston et al., 2013). The indication from the critical soil pH at 90% *RY* (Fig. 5) is that less P fertiliser is required at higher pH values. Due to a lack of data it was not possible predict the pH at 90% *RY* for all crops in the LTL experiment. Additional field experiments are needed to fill the gaps, especially for minor crops such as linseed, lupins and triticale. Likewise, there is insufficient understanding (or data) on the impact of soil pH on crop quality parameters, e.g. protein or grain nutrient for cereals and tuber quality for potato.

A further implication arising from the critical pH values found here (Fig. 5) is the difficulty of maintaining an optimal soil pH for a whole crop rotation. Because of the wide range in the sensitivity of crops to pH the target pH must suit all crops within a rotation. Walker et al. (2011) reported that the optimal pH was 5.5 for an eight course rotation of grass and arable crops (including cereals, potato and swedes) on a granite soil near Aberdeen, UK. This is a much lower pH than that usually considered critical for most crops in that study (compared with Fig. 5). In the UK the current recommendation for continuous arable cropping on mineral soils is to maintain a pH of 6.5 (AHDB, 2017). This is higher than that suggested by the LTL experiment as optimal for maintaining the yields of winter triticale, but too low for linseed (Fig. 5). The Nutrient Management Guide (RB209; AHDB, 2017) includes a note that “maintaining soil pH between 6.5 and 7.0 is justified for growing acid-sensitive crops such as sugar beet”. Thus, rotations which include acid tolerant crops such as triticale (Fig. 5), oats or potatoes are able to cope with a much lower critical soil pH. Critical soil pH values for a larger number of crops than were tested in this study have been published (MAFF, 1981). Additional field experiments are required in the future to evaluate the critical soil pH for all arable crops and update the pH values which are > 35 years old (MAFF, 1981).

A recent survey of arable soils in the UK showed that >40% have a soil pH < 6.5 (PAAG, 2015). This indicates that a large proportion of arable land is being maintained below the optimal soil pH and Goulding (2016) observed that the amount of lime applied to UK agricultural land is less than that required. Apart from reduced crop yields there are other implications for crop production from sub-optimal pH: e.g. some crop diseases are influenced by soil pH such as with clubroot (*Plasmodiophora brassicae*), while raising the pH can provide control (McGrann et al., 2016).

An improved understanding of the economic costs of liming compared to yield losses would further assist in determining the implications of maintaining the soil pH at the recommended optimum. For example, Tumusiime et al. (2011) calculated the effect of the cost of lime on setting N requirements. Indeed, there are many opportunities for further work on the LTL experiment at Rothamsted and Woburn. In the future analysis of the data presented here will be available via the electronic Rothamsted Archive, e-RA (www.era.rothamsted.ac.uk) (Perryman et al., 2018).

## 5 Conclusion

Although the general nature of *RY*-pH and yield-pH relationships are well known there has been a lack of specific detail for particular crops and soils. The Long-Term Liming experiment at Rothamsted and Woburn is invaluable in contributing to our understanding of arable yield response to liming. The quantification of the *RY*-pH relationships in this experiment demonstrates differences between crops in their critical pH and significant effects of site and, hence, soil type on *RY*-pH relationships for several crops. A significant P fertiliser x lime interaction effect was detected for selected crops: P input significantly reduced the predicted critical pH value for spring barley, winter triticale and winter wheat, but there was no P fertiliser effect for spring beans or linseed. For these cereal crops the addition of P (+P factor) increased the crop response to lime. Correspondingly, there was a decrease in the critical pH at 90% *RY* for soil with fertiliser P compared to the P control. Recent surveys have shown that a large area of arable soils in the UK are < pH 6.5 and there is an urgent need for further research on crop response to liming. This paper provides robust quantification of the *RY*-pH relationship for spring barley, but there is a need for additional investigation of the *RY*-pH and yield-pH relationship for other cereal (e.g. wheat, triticale, oats), oilseed and pulse crops. Moreover, further research is required on liming impacts on other aspects of crop response such as quality variables.
