# Corrigendum to “Yield responses of arable crops to liming – An evaluation of relationships between yields and soil pH from a long-term liming experiment” [Eur. J. Agron. 105 (2019) 176–188]

**DOI:** 10.1016/j.eja.2024.127221

**Published:** 2024-09

**Authors:** J.E. Holland, P.J. White, M.J. Glendining, K.W.T. Goulding, S.P. McGrath

**Affiliations:** a AFBI, Newforge lane, Belfast BT5 9PX, UK; b Intelligent Data Ecosystems, Rothamsted Research, Harpenden, Hertfordshire AL5 2JQ, UK; c Sustainable Soils and Crops, Rothamsted Research, Harpenden, Hertfordshire AL5 2JQ, UK

The authors regret an error in Table 3 of the above paper that gives the amount of Phosphate applied to the experiments. These are shown as kg P_2_O_5_ ha^−1^ for 1962–1978, but labelled as kg P ha^−1^ not P_2_O_5_ for 1980–1987 although the data are kg P_2_O_5_ ha^−1^. Below we provide a revised Table 3 with the correct data, all as kg P ha^−1^. The amounts of P applied were not used in the analyses of the data, so the error had no effect on the Results, Discussion and Conclusions.

**Table 3:** Phosphorus (P) applications to the Rothamsted and Woburn long-term liming experiments, 1962–1996.

Harvest year^a^		P applied (kg P ha^−1^ yr^−1^)			
**1962–1980:**		**Control (0)**		**+P**	
1962–1978^b^		0		27.5	
1968, 1974^c^		0		55.0	
**TOTAL 1962–1980: kg P ha**^−1^		**0**		**495**	
**1981–1996**^d^**:**	**Control (P0)**	**P1**	**P2**	**P3**	
1981	Rothamsted	0	25	25	75
	Woburn	0	25	25	75
1982	Rothamsted	0	50	0	50
	Woburn	0	50	0	50
1983	Rothamsted	0	0	50	50
	Woburn	0	50	50	100
1988	Rothamsted	0	25	25	75
	Woburn	0	25	25	75
**TOTAL 1981–1996: kg P ha**^−1^					
	**Rothamsted**	**0**	**100**	**100**	**250**
	**Woburn**	**0**	**150**	**100**	**300**

a P applied in the spring, except for Woburn 1964 and 1981, and Rothamsted 1981 and 1988, when applied the previous autumn.

b No P applied in the fallow years 1969, 1979, 1980.

c Higher P rate applied to potatoes in 1968 and 1974.

d From 1981 onwards the two P treatments (control, +P) were divided into four P treatments (control, P1, P2 and P3). Eight of the control plots were made into a new control (P0) and the other eight became a P1 treatment, similarly the +P plots were made into P2 and P3 treatments. P was applied in the four years indicated, with no P applied 1989–1996.

The authors would like to apologise for any inconvenience caused.

The correct data are also available from the Electronic Rothamsted Archive (e-RA https://www.era.rothamsted.ac.uk/), see Glendining (2020a, 2020b):

Glendining M.J. (2020a) Rothamsted long-term liming experiment lime and fertilizer treatments 1962–1996. Electronic Rothamsted Archive, Rothamsted Research, Harpenden UK. https://doi.org/10.23637/rcs10-Treatments-01

Glendining M.J. (2020b) Woburn long-term liming experiment lime and fertilizer treatments 1962–1996. Electronic Rothamsted Archive, Rothamsted Research, Harpenden UK. https://doi.org/10.23637/wcs10-Treatments-01

