# Supplementary material for: Yield responses of arable crops to liming – An evaluation of relationships between yields and soil pH from a long-term liming experiment
Source: Eur J Agron. 2019 Apr;105:176–88. doi: 10.1016/j.eja.2019.02.016 (PMC6472519; doi:10.1016/j.eja.2019.02.016)
Supplement: Supplementary file 1 [file mmc1.docx]

**Supplementary material**

**Table S1. The treatments applied and the number^a^ of treatment levels applied to whole and sub-plots for each year of the long-term liming experiment at Rothamsted and Woburn, 1962-1996**

| **Harvest year** | **Lime^b^** | **P^b^** | **K^b^** | **Mg^c^** | **Mn^c^** | **S^c^** | **Seed Inoculum^c^** |
| --- | --- | --- | --- | --- | --- | --- | --- |
| 1962 | 4 | 2 | 2 |  |  |  | 2 |
| 1963 | 4 | 2 | 2 |  |  |  |  |
| 1964 | 4 | 2 | 2 |  |  |  |  |
| 1965 | 4 | 2 | 2 |  |  |  |  |
| 1966 | 4 | 2 | 2 |  |  |  |  |
| 1967 | 4 | 2 | 2 |  |  |  |  |
| 1968 | 4 | 2 | 2 |  |  |  |  |
| 1969^d^ |  |  |  |  |  |  |  |
| 1970^e^ | 4 | 2 | 2 |  |  |  |  |
| 1971^e^ | 4 | 2 | 2 |  |  |  |  |
| 1972^e^ | 4 | 2 | 2 |  |  |  |  |
| 1973^e^ | 4 | 2 | 2 |  |  |  |  |
| 1974 | 4 | 2 | 2 | 2 |  |  |  |
| 1975 | 4 | 2 | 2 | 2 |  |  |  |
| 1976 | 4 | 2 | 2 | 2 |  |  |  |
| 1977 | 4 | 2 | 2 | 2 |  |  |  |
| 1978 | 4 | 2 | 2 | 2 |  |  |  |
| 1979^d^ |  |  |  |  |  |  |  |
| 1980^d^ |  |  |  |  |  |  |  |
| 1981 | 4 | 4 |  |  |  |  |  |
| 1982 | 4 | 4 |  |  |  |  |  |
| 1983 | 4 | 4 |  |  |  |  |  |
| 1984^d^ |  |  |  |  |  |  |  |
| 1985 | 4 | 4 |  |  |  |  |  |
| 1986 | 4 | 4 |  |  |  |  |  |
| 1987 | 4 | 4 |  |  | 2 |  |  |
| 1988 | 4 | 4 |  |  | 2 |  |  |
| 1989 | 4 | 4 |  |  | 2 |  |  |
| 1990 | 4 | 4 |  |  | 2 |  |  |
| 1991 | 4 | 4 |  |  |  | 2 |  |
| 1992 | 4 | 4 |  |  |  | 2 |  |
| 1993 | 4 | 4 |  |  |  | 2 |  |
| 1994 | 4 | 4 |  |  |  | 2 |  |
| 1995 | 4 | 4 |  |  |  | 2 |  |
| 1996 | 4 | 4 |  |  |  | 2 |  |

^a^ The number of levels for each treatment is given in brackets

^b^ Treatments applied to whole plots

^c^ Treatments applied to sub-plots

^d^ Fallow i.e. no crop grown

^e^ Four lime treatments but crop failed on some or all of the Control treatment which has thus been omitted from the analysis

**Table S2. The annual and growing season (April-July) total rainfall (mm) for each year during the long-term liming experiment at the Rothamsted and Woburn sites, 1962 - 1996**

| **Year** | **Rothamsted – total (mm)** | **Rothamsted – growing season (mm)** | **Woburn – total (mm)** | **Woburn -**  **growing season (mm)** |
| --- | --- | --- | --- | --- |
| 1962 | 646.5 | 178.0 | 558.4 | 163.0 |
| 1963 | 692.5 | 233.1 | 561.5 | 160.3 |
| 1964 | 526.6 | 253.7 | 457.3 | 201.5 |
| 1965 | 758.5 | 265.9 | 714.1 | 233.9 |
| 1966 | 790.3 | 304.4 | 830.3 | 301.9 |
| 1967 | 777.2 | 278.0 | 648.7 | 246.6 |
| 1968 | 758.2 | 255.5 | 699.9 | 291.8 |
| 1969 | 603.7 | 203.9 | 560.0 | 180.1 |
| 1970 | 710.9 | 170.3 | 600.3 | 153.2 |
| 1971 | 623.5 | 203.9 | 608.3 | 189.9 |
| 1972 | 638.5 | 163.9 | 493.7 | 183.2 |
| 1973 | 517.7 | 252.0 | 478.3 | 266.2 |
| 1974 | 865.9 | 153.7 | 788.8 | 158.2 |
| 1975 | 612.3 | 160.6 | 597.7 | 197.4 |
| 1976 | 575.0 | 99.1 | 496.6 | 81.3 |
| 1977 | 706.0 | 156.4 | 691.6 | 154.1 |
| 1978 | 724.4 | 266.6 | 620.1 | 221.6 |
| 1979 | 874.0 | 255.3 | 832.7 | 247.4 |
| 1980 | 610.6 | 199.1 | 724.4 | 257.7 |
| 1981 | 712.8 | 227.9 | 645.3 | 211.7 |
| 1982 | 796.6 | 237.3 | 679.4 | 204.8 |
| 1983 | 673.9 | 272.7 | 627.2 | 298.7 |
| 1984 | 749.8 | 152.9 | 682.9 | 157.3 |
| 1985 | 618.2 | 230.2 | 638.0 | 285.8 |
| 1986 | 762.5 | 206.2 | 715.9 | 208.8 |
| 1987 | 749.3 | 262.2 | 697.7 | 283.5 |
| 1988 | 705.4 | 230.3 | 668.7 | 243.9 |
| 1989 | 640.8 | 176.5 | 621.6 | 206.6 |
| 1990 | 597.2 | 94.5 | 460.5 | 97.8 |
| 1991 | 637.2 | 261.9 | 582.4 | 237.1 |
| 1992 | 844.2 | 264.9 | 815.5 | 266.7 |
| 1993 | 888.3 | 324.1 | 794.7 | 291.3 |
| 1994 | 725.9 | 175.3 | 609.9 | 161.0 |
| 1995 | 636.2 | 86.5 | 650.2 | 99.3 |
| 1996 | 515.4 | 110.3 | 469.3 | 123.0 |
| Mean | 693.3 | 210.5 | 637.8 | 207.6 |
| Minimum | 515.4 | 86.5 | 457.3 | 81.3 |
| Maximum | 888.3 | 324.1 | 832.7 | 301.9 |

**Table S3. The annual and growing season (April to July) total cumulative temperature (^o^C) for each year during the long-term liming experiment at the Rothamsted and Woburn sites, 1962 – 1996**

| **Year** | **Rothamsted – total (^o^C)** | **Rothamsted – growing season (^o^C)** | **Woburn – total (^o^C)** | **Woburn -**  **growing season (^o^C)** |
| --- | --- | --- | --- | --- |
| 1962 | 2987.6 | 1376.4 | 3004.0 | 1375.2 |
| 1963 | 2899.0 | 1459.5 | 2915.4 | 1461.7 |
| 1964 | 3331.1 | 1585.8 | 3357.4 | 1581.2 |
| 1965 | 3137.0 | 1427.3 | 3168.2 | 1427.9 |
| 1966 | 3341.4 | 1468.8 | 3355.7 | 1457.7 |
| 1967 | 3425.9 | 1487.7 | 3463.9 | 1467.9 |
| 1968 | 3291.6 | 1437.5 | 3352.3 | 1435.4 |
| 1969 | 3302.0 | 1488.2 | 3397.6 | 1524.6 |
| 1970 | 3339.8 | 1531.4 | 3458.2 | 1569.1 |
| 1971 | 3411.0 | 1461.6 | 3419.6 | 1441.1 |
| 1972 | 3278.5 | 1396.9 | 3295.4 | 1402.8 |
| 1973 | 3433.6 | 1490.5 | 3400.8 | 1470.8 |
| 1974 | 3401.2 | 1448.3 | 3377.0 | 1398.0 |
| 1975 | 3518.5 | 1502.3 | 3533.6 | 1501.5 |
| 1976 | 3592.9 | 1693.0 | 3618.0 | 1704.5 |
| 1977 | 3349.3 | 1351.5 | 3355.2 | 1342.5 |
| 1978 | 3332.1 | 1382.8 | 3347.8 | 1385.0 |
| 1979 | 3107.9 | 1450.1 | 3182.3 | 1471.0 |
| 1980 | 3265.8 | 1421.8 | 3346.3 | 1431.0 |
| 1981 | 3233.6 | 1417.6 | 3321.0 | 1457.7 |
| 1982 | 3485.0 | 1563.4 | 3552.2 | 1573.0 |
| 1983 | 3574.0 | 1549.8 | 3651.6 | 1550.4 |
| 1984 | 3429.2 | 1442.2 | 3503.1 | 1444.6 |
| 1985 | 3142.9 | 1442.5 | 3212.0 | 1461.2 |
| 1986 | 3107.2 | 1430.6 | 3207.6 | 1484.6 |
| 1987 | 3177.7 | 1457.6 | 3247.9 | 1490.5 |
| 1988 | 3455.3 | 1444.2 | 3545.2 | 1485.4 |
| 1989 | 3786.1 | 1580.2 | 3850.7 | 1580.2 |
| 1990 | 3749.6 | 1513.9 | 3879.5 | 1526.4 |
| 1991 | 3338.4 | 1403.6 | 3412.3 | 1458.3 |
| 1992 | 3578.5 | 1651.5 | 3591.2 | 1642.7 |
| 1993 | 3459.0 | 1574.2 | 3476.0 | 1571.0 |
| 1994 | 3750.8 | 1590.4 | 3751.6 | 1578.0 |
| 1995 | 3779.2 | 1619.5 | 3748.1 | 1622.2 |
| 1996 | 3292.6 | 1483.6 | 3280.7 | 1469.9 |
| Mean | 3373.8 | 1486.4 | 3416.5 | 1492.7 |
| Minimum | 2899.0 | 1351.5 | 2915.4 | 1342.5 |
| Maximum | 3786.1 | 1693.0 | 3879.5 | 1704.5 |

**Table S4. The annual and growing season (April to July) total cumulative solar radiation (MJ m^-2^) for each year during the long-term liming experiment at the Rothamsted and Woburn^a^ sites, 1962 – 1996**

| **Year** | **Rothamsted – total (MJ m^-2^)** | **Rothamsted – growing season (MJ m^-2^)** | **Woburn – total (MJ m^-2^)** | **Woburn -**  **growing season (MJ m^-2^)** |
| --- | --- | --- | --- | --- |
| 1962 | 3198.3 | 1776.2 | - | - |
| 1963 | 3108.3 | 1781.6 | - | - |
| 1964 | 3167.0 | 1743.9 | - | - |
| 1965 | 3076.6 | 1625.1 | - | - |
| 1966 | 3089.2 | 1715.4 | - | - |
| 1967 | 3247.2 | 1833.3 | - | - |
| 1968 | 2882.9 | 1687.1 | - | - |
| 1969 | 3098.5 | 1927.0 | - | - |
| 1970 | 3159.8 | 1738.5 | - | - |
| 1971 | 3145.9 | 1780.0 | - | - |
| 1972 | 2969.6 | 1641.1 | - | - |
| 1973 | 3306.3 | 1855.9 | - | - |
| 1974 | 3097.0 | 1819.3 | - | - |
| 1975 | 3271.5 | 1873.6 | - | - |
| 1976 | 3724.6 | 2159.8 | - | - |
| 1977 | 3347.5 | 1913.5 | - | - |
| 1978 | 3354.2 | 1790.2 | - | - |
| 1979 | 3471.6 | 1909.4 | - | - |
| 1980 | 3465.3 | 1932.7 | - | - |
| 1981 | 3165.4 | 1689.2 | - | 1775.3 |
| 1982 | 3489.0 | 1951.3 | - | - |
| 1983 | 3533.5 | 1963.0 | - | 1945.7 |
| 1984 | 3532.9 | 2095.5 | - | - |
| 1985 | 3365.0 | 1807.7 | - | - |
| 1986 | 3368.5 | 1812.1 | - | - |
| 1987 | 3264.4 | 1801.9 | - | - |
| 1988 | 3310.2 | 1771.2 | 3534.0 | 1874.2 |
| 1989 | 3745.5 | 2135.8 | 3757.5 | 2125.7 |
| 1990 | 3825.0 | 2136.0 | 3887.5 | 2131.1 |
| 1991 | 3349.8 | 1748.0 | 3372.8 | 1741.7 |
| 1992 | 3412.0 | 1993.7 | 3352.5 | 1928.5 |
| 1993 | 3420.3 | 1891.6 | 3058.9 | 1668.8 |
| 1994 | 3591.2 | 2062.3 | 3537.7 | 2018.4 |
| 1995 | 3850.8 | 2100.5 | 3731.6 | 2031.6 |
| 1996 | 3641.7 | 2084.1 | 3514.4 | 2013.0 |
| Mean | 3344.2 | 1872.8 | 3527.4 | 1948.1 |
| Minimum | 2882.9 | 1625.1 | 3058.9 | 1668.8 |
| Maximum | 3850.8 | 2159.8 | 3887.5 | 2131.1 |

^a^ Data only available after 1981

**Table S5. The significance level (*P* value)**^a^ **of lime, P and K treatment effects on soil pH for each crop of each year of the long-term liming experiment at Rothamsted, 1962-1996**

| **Year** | **Lime** | **P** | **K** | **Lime 🞨 P** | **Lime 🞨 K** | **P 🞨 K** | **Lime 🞨 P 🞨 K** |
| --- | --- | --- | --- | --- | --- | --- | --- |
| 1962^b^ | ns | ns | ns | ns | ns | ns | ns |
| 1963 | <0.001 | ns | ns | ns | ns | ns | ns |
| 1964 | <0.001 | ns | ns | ns | ns | ns | ns |
| 1965 | <0.001 | ns | ns | ns | ns | ns | ns |
| 1966 | <0.001 | ns | ns | ns | ns | ns | ns |
| 1967 | <0.001 | ns | ns | ns | ns | ns | ns |
| 1968 | <0.001 | ns | ns | ns | ns | ns | ns |
| 1970 | <0.001 | ns | ns | ns | ns | ns | ns |
| 1971 | <0.001 | ns | ns | ns | ns | ns | ns |
| 1972 | <0.001 | ns | ns | ns | ns | ns | ns |
| 1973 | <0.001 | ns | ns | ns | ns | ns | ns |
| 1974 | <0.001 | ns | ns | ns | ns | ns | ns |
| 1975 | <0.001 | ns | ns | ns | ns | ns | ns |
| 1978 | <0.001 | ns | ns | ns | ns | ns | ns |
| 1981 | <0.001 | ns | - | ns | - | - | - |
| 1982 | <0.001 | ns | - | ns | - | - | - |
| 1983 | <0.001 | 0.033 | - | ns | - | - | - |
| 1985 | <0.001 | 0.029 | - | ns | - | - | - |
| 1986 | <0.001 | ns | - | ns | - | - | - |
| 1987 | <0.001 | ns | - | ns | - | - | - |
| 1988 | <0.001 | ns | - | ns | - | - | - |
| 1989 | <0.001 | ns | - | ns | - | - | - |
| 1990 | <0.001 | ns | - | ns | - | - | - |
| 1991 | <0.001 | ns | - | ns | - | - | - |
| 1993 | <0.001 | ns | - | ns | - | - | - |
| 1994 | <0.001 | ns | - | ns | - | - | - |

^a^ ns indicates a *P* value > 0.05

^b^ 1962 soil pH measured before lime applied

**Table S6. The significance level (*P* value)**^a^ **of lime P and K treatment effects on soil pH for each crop of each year of the long-term liming experiment at Woburn, 1962-1996**

| **Year** | **Lime** | **P** | **K** | **Lime 🞨 P** | **Lime 🞨 K** | **P 🞨 K** | **Lime 🞨 P 🞨 K** |
| --- | --- | --- | --- | --- | --- | --- | --- |
| 1962^b^ | ns | ns | ns | ns | ns | ns | ns |
| 1963 | <0.001 | ns | ns | ns | ns | ns | ns |
| 1964 | <0.001 | ns | ns | ns | ns | ns | ns |
| 1965 | <0.001 | ns | ns | ns | ns | ns | 0.027 |
| 1966 | <0.001 | ns | ns | ns | ns | 0.04 | 0.015 |
| 1967 | <0.001 | ns | ns | ns | ns | 0.028 | 0.058 |
| 1968 | <0.001 | 0.028 | 0.014 | ns | ns | ns | ns |
| 1970 | <0.001 | 0.019 | ns | ns | ns | ns | ns |
| 1971 | <0.001 | ns | ns | ns | ns | ns | ns |
| 1972 | <0.001 | ns | ns | ns | ns | ns | ns |
| 1973 | <0.001 | 0.016 | ns | 0.005 | 0.008 | ns | 0.033 |
| 1974 | <0.001 | ns | ns | ns | ns | ns | ns |
| 1975 | <0.001 | ns | ns | 0.034 | 0.057 | ns | ns |
| 1978 | <0.001 | ns | ns | 0.012 | ns | ns | 0.024 |
| 1979 | <0.001 | ns | ns | ns | ns | ns | ns |
| 1980 | <0.001 | ns | ns | ns | ns | ns | ns |
| 1981 | <0.001 | 0.023 | - | 0.056 | - | - | - |
| 1982 | <0.001 | ns | - | ns | - | - | - |
| 1983 | <0.001 | ns | - | ns | - | - | - |
| 1985 | <0.001 | ns | - | ns | - | - | - |
| 1986 | <0.001 | ns | - | ns | - | - | - |
| 1987 | <0.001 | ns | - | ns | - | - | - |
| 1988 | <0.001 | ns | - | ns | - | - | - |
| 1991 | <0.001 | ns | - | ns | - | - | - |
| 1994 | <0.001 | ns | - | ns | - | - | - |

^a^ ns indicates a *P* value > 0.05

^b^ 1962 soil pH measured before lime applied

**Table S7. The significance level (*P* value)**^a^ **of lime and P and the interaction (lime 🞨 P) treatment effects on soil extractable P (Olsen) for selected years (between 1962 and 1996) during the long-term liming experiment at Rothamsted and Woburn**

| **Year** | **Rothamsted**  **Lime** | **P** | **Lime 🞨 P** | **Woburn**  **Lime** | **P** | **Lime 🞨 P** |
| --- | --- | --- | --- | --- | --- | --- |
| 1968^b^ | ns | <0.001 | ns | - | - | - |
| 1972^b^ | 0.026 | <0.001 | ns | - | - | - |
| 1973 | ns | <0.001 | ns | <0.001 | <0.001 | ns |
| 1981 | ns | <0.001 | ns | <0.001 | <0.001 | ns |
| 1982 | 0.039 | <0.001 | ns | <0.001 | <0.001 | 0.026 |
| 1986 | 0.018 | <0.001 | ns | <0.001 | <0.001 | 0.009 |
| 1989 | 0.001 | <0.001 | ns | <0.001 | <0.001 | ns |
| 1994 | 0.006 | <0.001 | ns | <0.001 | <0.001 | 0.025 |

^a^ ns indicates a *P* value > 0.05

^b^ No plot level data for Woburn

**Table S8. The effect of lime and K fertiliser treatments on mean soil exchangeable K (mg 100 g^-1^ soil) for selected years (between 1962 and 1996) during the long-term liming experiment at Rothamsted and Woburn**

| **Year** | **Site** | **K treatment** | **Control (lime)** | **Low** | **Medium** | **High** | **SED** |
| --- | --- | --- | --- | --- | --- | --- | --- |
| 1964 | Rothamsted | control-K | 664 | 592 | 690 | 686 | 153.8 |
| 1964 | Rothamsted | plus-K | 1062 | 888 | 489 | 930 |  |
| 1967 | Rothamsted | control-K | 624 | 592 | 613 | 510 | 92.9 |
| 1967 | Rothamsted | plus-K | 1549 | 1554 | 1529 | 1486 |  |
| 1971 | Rothamsted | control-K | 738 | 583 | 642 | 700 | 71.6 |
| 1971 | Rothamsted | plus-K | 1856 | 1756 | 1822 | 1783 |  |
| 1972 | Rothamsted | control-K | 706 | 546 | 530 | 568 | 65.6 |
| 1972 | Rothamsted | plus-K | 1952 | 1561 | 1666 | 1590 |  |
| 1989^a^ | Rothamsted |  | 1388 | 1298 | 1342 | 1316 | 338.8 |
| 1994^a^ | Rothamsted |  | 1335 | 1366 | 1508 | 1360 | 130.2 |
| 1963 | Woburn | control-K | 277.8 | 260 | 254.8 | 263 | 9.22 |
| 1963 | Woburn | plus-K | 387.2 | 364.8 | 347 | 353.8 |  |
| 1964 | Woburn | control-K | 584 | 523 | 530 | 525 | 27.2 |
| 1964 | Woburn | plus-K | 930 | 844 | 871 | 854 |  |
| 1967 | Woburn | control-K | 446 | 464 | 460 | 432 | 25.5 |
| 1967 | Woburn | plus-K | 1314 | 1184 | 1104 | 1096 |  |
| 1971 | Woburn | control-K | 484 | 479 | 478 | 427 | 44.8 |
| 1971 | Woburn | plus-K | 1290 | 1086 | 1064 | 934 |  |
| 1972 | Woburn | control-K | 466 | 378 | 402 | 375 | 29.2 |
| 1972 | Woburn | plus-K | 1493 | 1266 | 1387 | 1216 |  |
| 1973 | Woburn | control-K | 530 | 428 | 468 | 440 | 44.4 |
| 1973 | Woburn | plus-K | 1538 | 1468 | 1435 | 1415 | 94.8 |
| 1989^a^ | Woburn |  | 954 | 1037 | 976 | 1062 |  |

^a^From 1981 onwards there was no K main plot treatment

**Table S9. The significance level (*P* value)**^a^ **of lime and K and the interaction (lime 🞨 K) treatment effects on soil exchangeable K for selected years (between 1962 and 1996) during the long-term liming experiment at Rothamsted and Woburn**

| **Year** | **Rothamsted**  **Lime** | **K** | **Lime 🞨 K** | **Woburn**  **Lime** | **K** | **Lime 🞨 K** |
| --- | --- | --- | --- | --- | --- | --- |
| 1963^b^ | - | - | - | ns | <0.001 | ns |
| 1964 | ns | 0.021 | 0.043 | ns | <0.001 | ns |
| 1967 | ns | <0.001 | ns | 0.018 | <0.001 | 0.022 |
| 1971 | ns | <0.001 | ns | 0.03 | <0.001 | ns |
| 1972 | 0.024 | <0.001 | ns | <0.001 | <0.001 | ns |
| 1973^b^ | - | - | - | ns | <0.001 | ns |
| 1989^c^ | ns | - | - | ns | - | - |
| 1994^cd^ | ns | - | - | - | - | - |

^a^ ns indicates a *P* value > 0.05

^b^ No plot level data for Rothamsted

^c^ No K main plot treatment in 1989 or 1994, lime effect only

^d^ No plot level data for Woburn

**Table S10. The significance level (*P* value)^a^ for the treatment interactions for lime, P and K treatments for crop yield in each harvested year of the long-term liming experiment at Rothamsted and Woburn, 1962-1996**

| **Harvest year** | **Crop** | **Rothamsted**  **Lime 🞨 P** | **Lime 🞨 K^a^** | **P 🞨 K^b^** | **Lime 🞨 P 🞨 K^b^** | **Woburn**  **Lime 🞨P** | **Lime 🞨 K^a^** | **P 🞨 K^b^** | **Lime 🞨 P 🞨 K^b^** |
| --- | --- | --- | --- | --- | --- | --- | --- | --- | --- |
| 1963 | Spring beans | ns | ns | ns | ns | ns | ns | ns | ns |
| 1964 | Spring beans | ns | ns | ns | ns | ns | ns | 0.003 | 0.034 |
| 1965 | Spring barley | ns | ns | ns | ns | 0.019 | ns | ns | ns |
| 1966 | Spring barley | ns | ns | ns | ns | ns | ns | 0.010 | ns |
| 1967 | Spring barley | ns | ns | ns | ns | 0.045 | ns | ns | ns |
| 1968 | Potatoes | ns | ns | ns | ns | 0.031 | ns | 0.006 | ns |
| 1970 | Spring barley | ns | ns | ns | ns | 0.002 | ns | 0.022 | 0.016 |
| 1971 | Spring barley | ns | ns | ns | ns | 0.042 | <0.001 | ns | ns |
| 1972 | Spring barley | ns | ns | ns | ns | ns | ns | 0.040 | ns |
| 1973 | Spring barley | 0.043 | ns | ns | ns | ns | ns | ns | ns |
| 1974 | Potatoes | ns | 0.015 | ns | ns | 0.014 | <0.001 | <0.001 | ns |
| 1975 | Spring oats | ns | ns | ns | ns | ns | ns | ns | ns |
| 1977 | Spring oats | ns | ns | ns | ns | 0.001 | ns | 0.002 | 0.002 |
| 1978 | Spring barley | ns | ns | ns | ns | ns | ns | ns | ns |
| 1981 | Spring oats | ns | - | - | - | ns | - | - | - |
| 1982 | Spring oats | 0.05 | - | - | - | ns | - | - | - |
| 1983 | Potatoes | ns | - | - | - | 0.001 | - | - | - |
| 1985 | Spring barley | ns | - | - | - | ns | - | - | - |
| 1986 | Winter triticale | 0.002 | - | - | - | ns | - | - | - |
| 1987 | Spring lupins | ns | - | - | - | ns | - | - | - |
| 1988 | Linseed | ns | - | - | - | ns | - | - | - |
| 1989 | Spring beans | ns | - | - | - | ns | - | - | - |
| 1990^c^ | Spring beans | ns | - | - | - | - | - | - | - |
| 1991 | Winter oilseed rape | ns | - | - | - | 0.027 | - | - | - |
| 1993^c^ | Winter lupins | ns | - | - | - | - | - | - | - |
| 1995 | Winter wheat | ns | - | - | - | ns | - | - | - |
| 1996 | Winter wheat | ns | - | - | - | ns | - | - | - |

^a^ ns indicates a *P* value > 0.05

^b^ From 1981 onwards there was no K main plot treatment

^c^ Crop failure at Woburn only
